# Barriers and enablers of integrated care in the UK: a rapid evidence review of review articles and grey literature 2018–2022

**DOI:** 10.3389/fpubh.2023.1286479

**Published:** 2024-01-04

**Authors:** Linda J. M. Thomson, Helen J. Chatterjee

**Affiliations:** Department of Biosciences and Arts & Sciences, University College London, London, United Kingdom

**Keywords:** barriers, enablers, health and social care, integrated care, mental health, multiple morbidities, personalized care

## Abstract

Integrated care refers to person-centered and coordinated, health and social care, and community services. Integrated care systems are partnerships of organizations that deliver health and care services which were placed on a statutory footing in England, April 2022. Due to the need for fast, accessible, and relevant evidence, a rapid review was conducted according to World Health Organization methods to determine barriers and enablers of integrated care across the United Kingdom, 2018–2022. Nine databases were searched for review articles reporting evaluation of integrated care interventions involving medical (clinical and diagnostic) and nonmedical (public health services and community-based or social care/person-centred care) approaches, quality checked with the Critical Appraisal Skills Program qualitative checklist. OpenGrey and hand searches were used to identify grey literature, quality checked with the Authority, Accuracy, Coverage, Objectivity, Date, and Significance checklist. Thirty-four reviews and 21 grey literature reports fitted inclusion criteria of adult physical/mental health outcomes/multiple morbidities. Thematic analysis revealed six themes (collaborative approach; costs; evidence and evaluation; integration of care; professional roles; service user factors) with 20 subthemes including key barriers (cost effectiveness; effectiveness of integrated care; evaluation methods; focus of evidence; future research; impact of integration) and enablers (accessing care; collaboration and partnership; concept of integration; inter-professional relationships; person-centered ethos). Findings indicated a paucity of robust research to evaluate such interventions and lack of standardized methodology to assess cost effectiveness, although there is growing interest in co-production that has engendered information sharing and reduced duplication, and inter-professional collaborations that have bridged task-related gaps and overlaps. The importance of identifying elements of integrated care associated with successful outcomes and determining sustainability of interventions meeting joined-up care and preventive population health objectives was highlighted.

## 1 Introduction

Integrated care refers to “person-centered coordinated care” [(1), p. 3] and “care that is planned with people who work together to understand the service user and their carer(s), puts them in control and coordinates and delivers services to achieve the best outcomes” [(2), p. 1]. Integrated care systems (ICSs) have been described as “partnerships of organizations that come together to plan and deliver joined up health and care services, and to improve the lives of people who live and work in their area” [(3), p. 1]. Although ICSs were only placed on a statutory footing in England in April 2022 with a view to integrating health and social care services, the notion of integrated care has been promoted by practitioners and policy makers for around 60 years, as exemplified by the statement “The road ahead for preventive medicine seems clear. It is the delivery of high quality, personalized (as opposed to depersonalized) comprehensive medical care to all” [(4), p. 1]. For more than a decade, NHS England has administered three national pilot integrated care programs operated as voluntary partnerships with the objectives of “improving the clinical and cost effectiveness of care by removing duplication, avoiding care gaps and improving patient/user and informal carer experience” [(5), p. 1]. The authors found that although the pilots made headway in their objectives, they made little impact on unplanned hospital admissions. Despite general agreement on the aims of integrated care, there have been competing definitions of integration (6) and the lack of shared understanding of the term “integrated care” could result in different practices and priorities (5). Most authors, however, advocate definitions that are concerned with bringing organizations and professional service providers together and improving patient outcomes, for example, “ultimately, these integrated teams – rooted in the community and working across the spectrum of health and care – are the central conduit through which we can deliver the new model of integrated care” [(7), p. 8] and:

Integration is a coherent set of methods and models on the funding, administrative, organizational, service delivery and clinical levels designed to create connectivity, alignment and collaboration within and between the cure and care sectors. The goal of these methods and models is to enhance quality of care and quality of life, consumer satisfaction and system efficiency for patients with complex, long-term problems cutting across multiple services, providers and settings [(8), p. 3].

The devolved nations of the United Kingdom (UK) all implemented forms of integrated care prior to its inception in England. Wales set up its Integrated Care Fund (ICF) in 2014 which aimed to enable integrated working between social services, health, housing, and third sector and independent providers to “improve the lives of the most vulnerable people” in society [(9), p. 4]. The focus of Welsh integration was on anticipatory and preventative care and to develop sustainable services which “… helped services move away from some of the more traditional forms of patient care, including in hospital care, and has instead supported projects that are more person centered with care provided at or closer to home” [(9), p. 7]. The ICF initially focused on supporting older people, particularly those with long term conditions, and has extended this support over time to include other population groups such as children with complex needs or on the edge of care, autistic people, people with dementia, people with learning disabilities, and unpaid or young carers. Seven statutory regional partnership boards spanning 22 local authorities established in 2016 used ICF funding to help drive the integration of health, social care, and housing. The ICF noted that “while larger scale, strategic projects can have significant levels of positive impact on larger numbers of service users, smaller projects with lower levels of investment can equally have significant positive impacts… and also provide a useful smaller scale test bed from which successful delivery aspects can grow and be upscaled for future years” [(10), p. 22]. Saying that, the same report also commented that the Minister for Health and Social Services brought partners together in 2020 to reflect on what was working and what could be done “to improve shared learning and the challenges around mainstreaming” [(10), p. 38]. Changes introduced to address the recommendations of Audit Wales included a review of the governance around the ICF to ensure that appropriate scrutiny arrangements were in place for decisions made by regional partnership boards, and the mapping out funding streams across health and social care to ensure better alignment of the funding and to help partners take a more strategic approach to deploying collective resources.

Scotland legislated in 2016 to bring together health and social care creating 31 integration joint boards (IJBs) responsible for providing funding for local services previously managed by NHS Boards or local authorities (11). The reason for integration of health and social care was to “ensure people have access to the services and support they need, so that their care feels seamless to them, and so that they experience good outcomes and high standards of support” [(11), p. 2]. The Convention of Scottish Local Authorities’ review found that integration authorities operated in “an extremely challenging environment” and that their focus was on “tackling the challenges rather than revisiting the statutory basis for integration” [(11), p. 2]. These challenges included how IJBs operated and how services could be planned and delivered to ensure better outcomes. The review proposed that effective approaches for community engagement and participation needed to be put in place; that an improved understanding of working relationships with carers, people using the services and local communities was required; and that these people needed to be supported better.

Uniquely within the UK, Northern Ireland (NI) has had a structurally integrated system of health and social care since 1973 but documentation noted that:

Significantly, the original decision was not informed by theoretical models of health care, but by an urgent need to reorganize the system of local government, which had become widely discredited. There was little awareness that this model of reorganization, which was given a cautious, lukewarm welcome by health care professionals, would become viewed by many policymakers, politicians and academics as the Holy Grail [(12), p. 2].

In NI, 17 integrated care partnerships (ICPs) form a collaborative network of service providers including healthcare professionals such as doctors, nurses, pharmacists, social workers, and hospital specialists; the voluntary, community and social enterprise (VCSE) sector; local council representatives; and service users and carers (13). The ICPs work across five local commissioning group areas to ensure coverage of all general practices. The function of ICPs is to “support the vision to make home and the community the hub of care. They also aim to ensure that services are personalized and seamless; empower patients; promote health; and prevent illness, where possible” [(13), p. 1]. Towards the start of COVID-19 (April 2020), the ICP plan was put on hold to focus on the response to the pandemic. ICP leaders claimed to develop a joined-up approach to shielding individuals who could be referred to community support hubs, primary care, and community pharmacists. The Health and Social Care Board and the Public Health Agency administered the regional commissioning agenda through the integrated service teams underpinned by five local commissioning groups. Local Commissioning Groups are committees of the Health and Social Care Board responsible for ensuring that health and social care needs of local populations across NI are addressed. Local Commissioning Groups align geographically with the five Health and Social Care Trusts that provide services directly to the community. The Health and Social Care Board closed on 31 March 2022 with its functions transferred to the NI Department of Health as the Strategic Planning and Performance Group.

Prior to Brexit and just prior to the COVID-19 outbreak, NI was involved in the European VIGOUR project addressing key questions on how to put integrated care into practice, along with 14 regions of six other European countries: Austria (Styria); Greece (Crete); Italy (Campania, Friuli Venezia Giulia, Emilia Romagna, Lazio, Liguria, Piedmont, Trento, and Veneto); Netherlands (Twente); Poland (Lodz); Spain (Andalusia and Valencia) (14). The project, funded by the Third European Health Program, aimed to support care authorities in progressing the transformation of their health and care systems to provide sustainable models for integrated care. The integrated care support program set out to identify good practice within local circumstances and facilitate capacity building and upscaling. To achieve these aims, the project implemented a twinning scheme whereby VIGOUR pioneer care authorities were brought together for knowledge exchange activities (15). Other European countries have also endeavored to implement integrated care. Finland, for example, traditionally organized its primary care services around its 309 municipalities and population of 5.5 million people. An attempt to reform these services led to the collapse of the government in 2019 because some elements of the new legislation appeared to conflict with constitutional law (16). The current government in power since then has proposed new legislation to introduce 21 wellbeing service counties to create new regional identities, with the capital, Helsinki, organizing its own health and social care services (17). The proposed counties would have fewer responsibilities outside of health and social care services and, as patient choice and competition between public services were areas of contention under the previous government, they have been omitted from the legislation. It is interesting that the authors observed:

While all of this has been going on, local initiatives have been getting on with the practice of integrated person-centered care. There is nothing in the current legislation that stops local areas from acting collectively in this way. But as the progress has been slow and incremental, policy makers recognize the important role that legislating can play in signaling to the entire system the urgency and need for change [(17), p. 7].

Outside of Europe, though with a similarly low population density as Finland, the state of Oregon in the northwest of the United States of America (USA) with 4.2 million people, has been at the forefront of health reform for over 30 years. After passing the Affordable Care Act in 2010, the state legislated in 2011 to integrate health and social care systems creating 15 new coordinated care organizations (CCOs). The Oregon Legislative Assembly found that “achieving its goals of improving health, increasing the quality, reliability, availability and continuity of care and reducing the cost of care requires an integrated and coordinated health care system” [(18), p. 1]. CCOs coordinate benefits and services including non-health services, such as housing, food security and employment support; they follow standards for safe and effective care; and are locally accountable for health resource allocation. Their budget is fixed to counter the potential risk of overly high costs that occurred previously and CCOs are encouraged to make efficiency savings. In making savings though, CCOs run the risk of a reduction in their budgets for the following year. It is interesting that the majority of members on the CCO governing board need to be representatives of organizations that share the financial risk in addition to those from major health delivery systems. CCOs are also responsible for convening community advisory councils to ensure a community perspective (17). The authors point out, however, that one part of the system not included in CCOs is public health and that “this fissure was badly exposed during the Covid-19 epidemic, with a lack of coordination and planning between CCOs and local public health departments” [(17), p. 10].

In England, integrating health and social care was described as “an objective of national policy for more than three decades” that “started to gather pace with the introduction of the Health and Care Bill, which is expected to put ICSs on a statutory footing from April 2022” [(19), p. 1] now in place. Furthermore, the NHS long-term plan was seen to have reinforced the role of ICSs in establishing more collaborative working and joined-up care for the patients (20). There are currently 42 ICSs across England; each has an integrated care board (ICB) responsible for NHS and wider integration, and an integrated care partnership (ICP) responsible for promoting health, care and wellbeing. Each ICS also comprises partnerships at place level and joint arrangements at locality level through primary care networks. ICBs take on the planning functions previously held by clinical commissioning groups created by the 2012 Health and Social Care Act (19). Each ICB will be required to produce a five-year plan for how NHS services will be delivered taking into account the integrated care strategy setting out how the wider health needs of the local population should be met (19). The ICPs are statutory committees with the remit of integrating the NHS, local authorities, social care and other organizations as equal partners to focus more broadly on health, public health and social care, and to determine how the wider health needs of the local population should be addressed. Integrated care consequently involves “bringing organizations and professionals together, with the aim of improving outcomes for patients and service users” [(6), p. 3] where the intended outcomes might range from broad lifestyle goals to individualized care. Organizational integration alone, however, was viewed as “unlikely to deliver better outcomes” without additional efforts to “focus on clinical and service integration” [(6), p. 7]. ICSs were tasked with the broad aims of “improving outcomes in population health and healthcare; tackling inequalities in outcomes, experience and access; enhancing productivity and value for money; and helping the NHS support broader social and economic development” [(7), p. 4]. Some of these intended outcomes, however, were considered “not easily measurable” given that the criteria against which success is measured may “vary widely” and that the different target populations, intervention group sizes, and contexts may be difficult to compare [(6), p. 8].

The current review examined published review articles from 2018–2022 that employed a variety of review methods to measure and report outcomes of integrated care in the UK. The aims were to conduct a rapid review of evidence to determine the barriers and enablers of integrating health and social care and community resources, and to ascertain the extent of this evidence.

## 2 Methods

### 2.1 Rapid evidence review

A rapid evidence review was conducted based on established World Health Organization rapid review methods (21) and in keeping with a review of methodologies for evidence-informed decision making in health policy and practice (22) due to the need for fast, accessible, and relevant evidence to address the research objectives. A comprehensive search strategy was implemented for review articles of integrated care in the UK from January 2018 to December 2022; 2018 was chosen as the start date because in that year, the NHS selected and named areas of England to be the first ICSs to work closely with NHS England to pioneer best practice. The following search terms were used: [integrated care] [inter-organizational healthcare] [inter-professional collaboration] [co-production in healthcare] [comprehensive care] [collaborative care] [person-centered care] [personalized care]. Further variants of these search terms produced no new results. Searches were carried out in: Embase; Global Health; Google Scholar; PsychARTICLES; PsychINFO; PubMed; Science Direct; Scopus; and Web of Science producing 26,100 results.

Using clear inclusion and exclusion criteria (below) to screen titles and abstracts, the number of articles was reduced to 120 and full texts were scrutinized in more detail for fit to criteria leading to inclusion of 34 articles. Further hand searches were carried out after consulting references sections of selected articles but no further reviews fitting the criteria were located that did not duplicate those already selected. Using the above dates and search terms, 125 grey literature resources in the public domain were located in the OpenGrey database and by hand searching; screening and further scrutiny led to inclusion of 21 grey literature resources. Study selection and data extraction was conducted by one researcher and checked by a second consistent with rapid reviews.

Inclusion criteria comprised peer-reviewed quantitative, qualitative, or mixed methods articles and grey literature reports reviewing implementation or evaluation of integrated care interventions or programs for physical and/or mental health outcomes, and multiple morbidities in the adult population of one or more UK nations. Exclusion criteria comprised review articles or reports solely defining the concept of integrated care, the legislation behind it, or guidance to its implementation, with a single disease or COVID-19 focus, published in a language other than English, only considering healthcare transformation via digital technology and/or estate management, and lacking inclusion of evidence from one or more UK nations.

### 2.2 Data analysis

**Figure 1:**
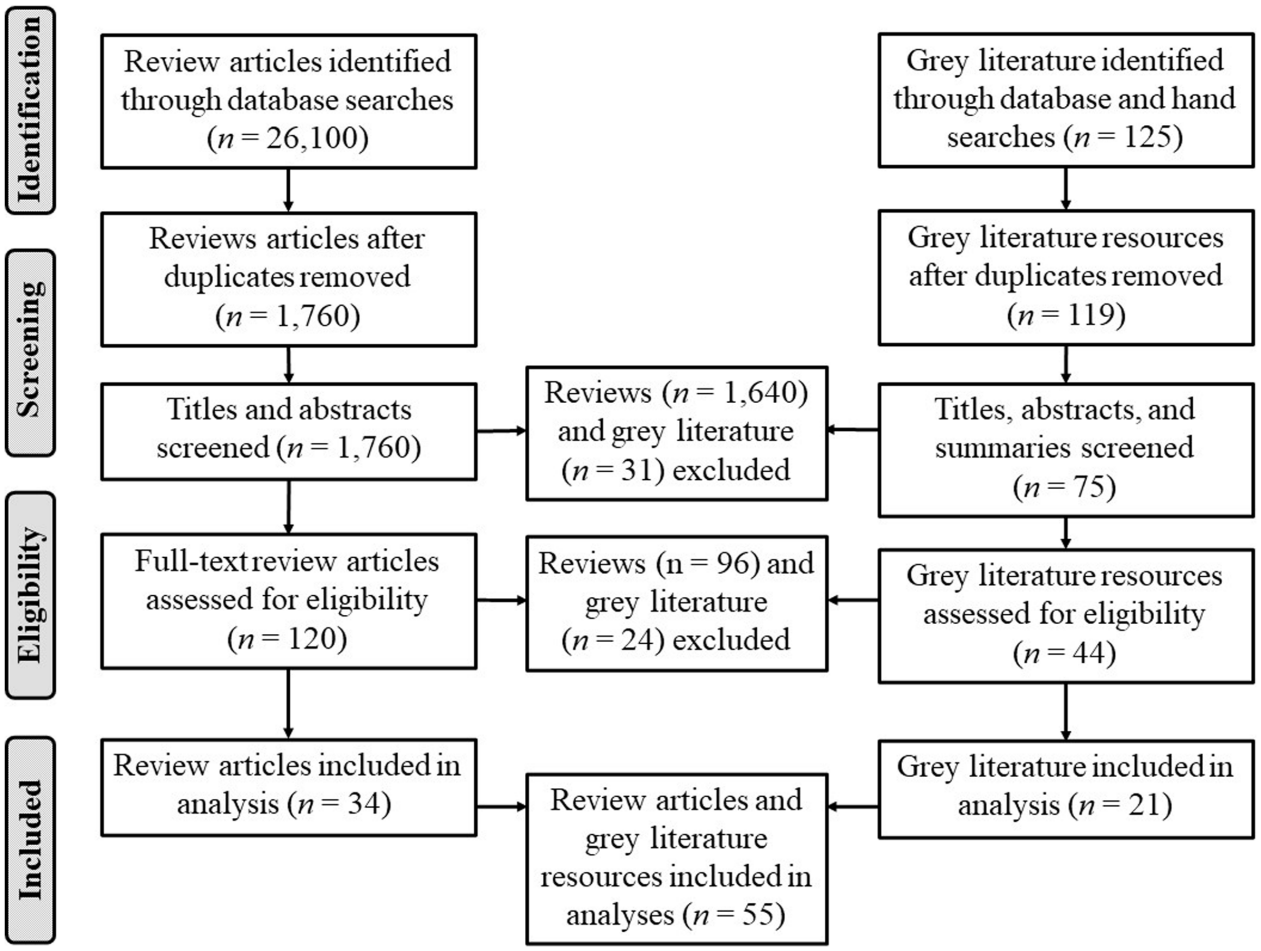
Review articles and grey literature: PRISMA flow diagram.

The review process adhered to the Preferred Reporting System for Meta-Analysis (PRISMA) (23) flow diagram (Figure 1) although the process of meta-analysis was not implemented as the reported resources were heterogeneous in terms of population, methods and outcomes tested. Similarly, AMSTAR was not used as it more suitable for systematic reviews. Instead, the Critical Appraisal Skills Program Qualitative Checklist tool (CASP) was applied to ensure review article quality (24). CASP’s ten questions concerning aims, methodology, research design, recruitment strategy, data collection, potential bias, ethics, rigor, findings, and value have a possible response set of “yes”, “cannot tell” or “no”. As the publications reviewed were review articles, the recruitment strategy question was modified to reflect the inclusion strategy of the articles reviewed. Although CASP does not have a scoring system due to its initial design as an educational pedagogic tool, articles were checked for successful fit to all questions. Similarly, grey literature reports were critically appraised for inclusion using the Authority, Accuracy, Coverage, Objectivity, Date, and Significance (AACODS) checklist (25). Thematic and content analyses were conducted in QSR software NVivo 12.7. Thematic analysis used an inductive approach to summarize the outcomes from which subthemes and themes were derived. Content analysis was employed to calculate the relative frequency of barriers and enablers of integrated care across the themes through the quantification of the summarized outcomes. Methodological triangulation was used to combine the proven methods of qualitative text analysis with quantitative frequency analysis to develop a comprehensive understanding of the outcomes that would not necessarily be evidenced using only one approach.

## 3 Results

Review articles (*n* = 34) and grey literature resources (*n* = 21) meeting inclusion criteria were tabulated and synthesized using a narrative summary of outcomes. Included review articles (Supplementary Table S1) jointly reviewed 2,037 publications (mean = 60; range = 265) from 1990–2021 consisting of peer-reviewed primary research quantitative, qualitative, and mixed methods studies, with ten (29%) incorporating grey literature; 14 articles were reviewed systematically; nine involved scoping; four employed a realist approach; three reviewed the literature, two were narrative syntheses; one was a mapping review; and one was a bibliometric analysis. Included grey literature resources (Supplementary Table S2) incorporated material from 2000–2022 and comprised 12 reports including three annual reports; three guides including a practical guide and statutory guidance; two reviews of 115 and 14 peer-reviewed and grey literature publications; two evidence briefings, one evaluation and one handbook.

Thematic analysis identified six themes and 20 subthemes comprising: (i) Collaborative Approach (subthemes: collaboration and partnership; co-production; inter-professional relationships), (ii) Costs (subthemes: cost effectiveness; cost savings), (iii) Evidence and Evaluation (subthemes: evaluation methods; findings; focus of evidence; future research), (iv) Integration of Care (subthemes: concept of integration; effectiveness of integrated care; impact of integration), (v) Professional Roles (subthemes: community stakeholders; employment and training; leadership; link worker role; professional identity), and (vi) Service User Factors (subthemes: accessing care; person-centered ethos).

**Figure 2:**
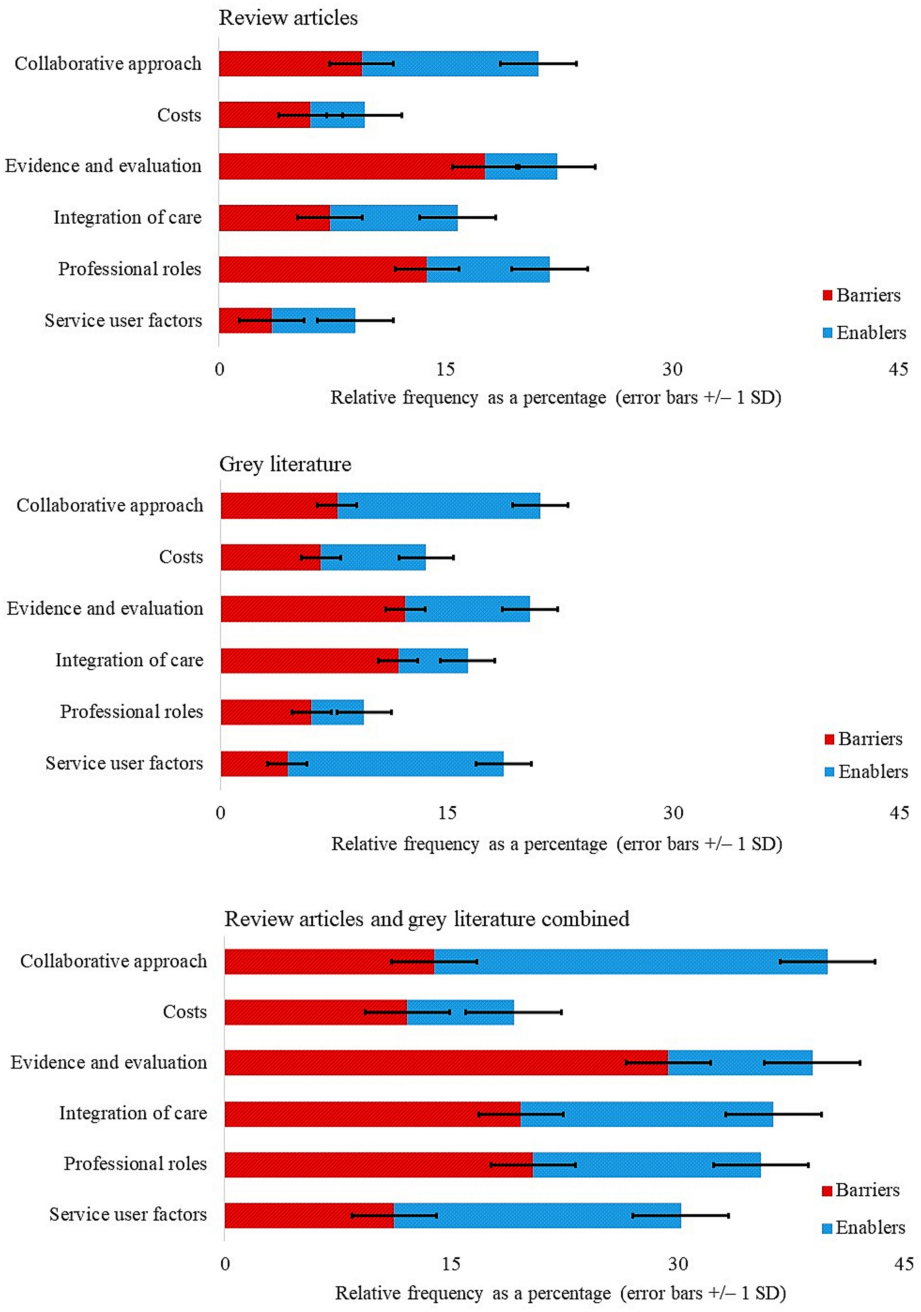
Relative frequency of barriers and enablers split by themes.

**Figure 3:**
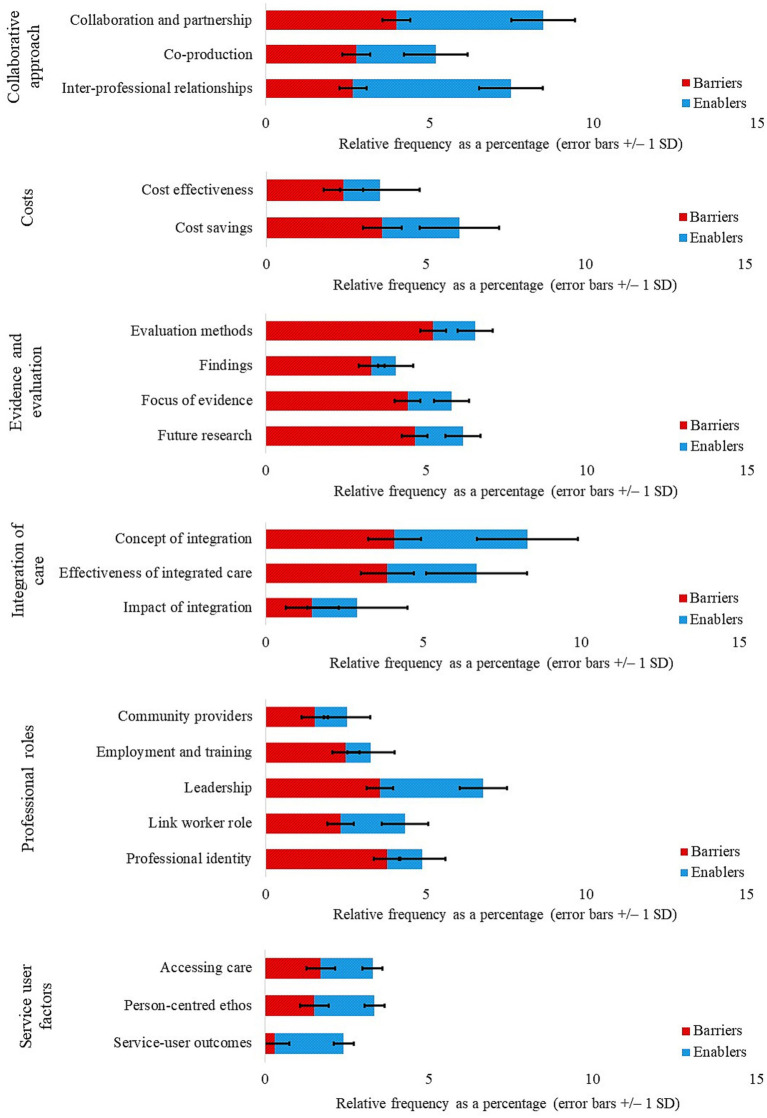
Review articles: subthemes split by barriers and enablers.

**Figure 4:**
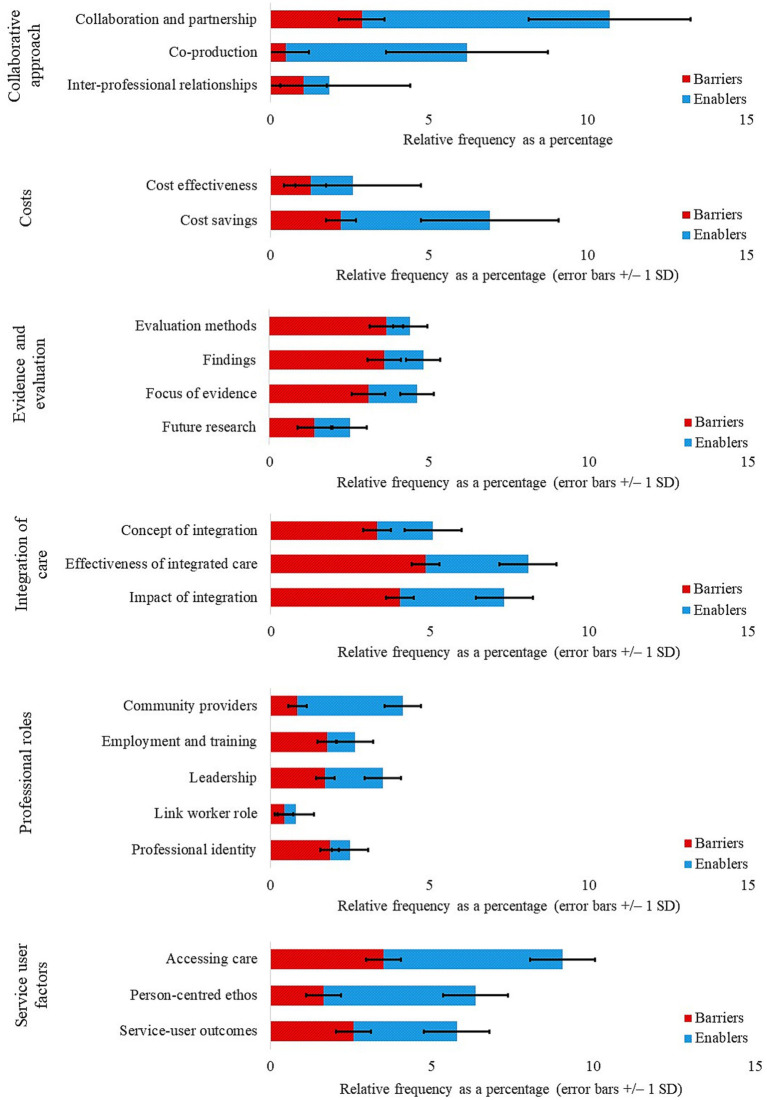
Grey literature: subthemes split by barriers and enablers.

**Figure 5:**
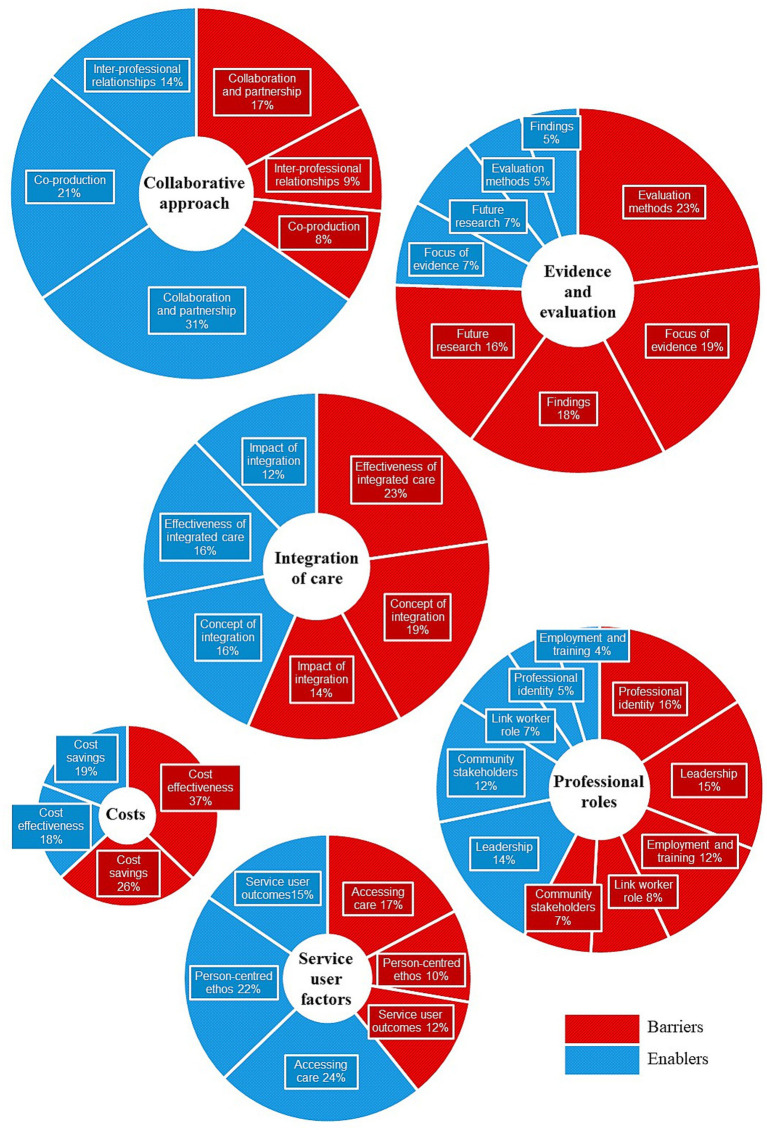
Review articles and grey literature combined: themes in proportion to frequency of responses with subthemes split by barriers and enablers.

Content analysis was employed to calculate the relative frequency of barriers and enablers of integrated care across themes derived from review articles and grey literature, both separately and combined (Figure 2). Barriers and enablers were further split across subthemes for review articles (Figure 3) and grey literature separately (Figure 4) and combined (Figure 5). For review articles the relative frequency of barriers was 57.70% and enablers was 42.30% (Supplementary Table S3) and for grey literature, the relative frequency of barriers was 48.87% and enablers was 51.13% (Supplementary Table S4).

Subthemes with frequencies in the upper quartile (4.06–7.81%) were regarded as key barriers and enablers (Table 1). Key barriers lay within the themes of Evidence and Evaluation (subthemes: evaluation methods; focus of evidence; future research) for review articles, and Costs (subtheme: cost effectiveness) and Integration of Care (subthemes: effectiveness of integrated care; impact of integration) for grey literature. Key enablers lay within the themes of Collaborative Approach (subthemes: collaboration and partnership; inter-professional relationships) and Integration of Care (subtheme: concept of integration) for review articles, and Collaborative Approach (subthemes: collaboration and partnership; co-production) and Service User Factors (subthemes: accessing care; person-centered ethos) for grey literature. Subthemes are addressed in turn under their respective themes with associated quotations from the reviewed resources.

**Table 1:** Relative frequency of barriers and enablers split by themes and subthemes.

Themes	Subthemes	Barriers/Enablers	Relative frequency as a percentage		Total subthemes		
			Review articles	Grey literature			
Collaborative Approach	Collaboration and partnership	Barriers	3.99	2.89	6.88		
		Enablers	4.48	7.81	12.29		
	Co-production	Barriers	2.77	0.50	3.27		
		Enablers	2.42	5.69	8.11		
	Inter-professional relationships	Barriers	2.67	1.05	3.72		
		Enablers	4.82	0.82	5.64		
	Total subthemes	Barriers	**9.43**	**4.44**	**13.87**		
		Enablers	**11.72**	**14.32**	**26.04**		
Costs	Cost effectiveness	Barriers	2.42	4.69	7.11		
		Enablers	1.14	2.23	3.37		
	Cost savings	Barriers	3.64	1.33	4.97		
		Enablers	2.42	1.27	3.69		
	Total subthemes	Barriers	**6.06**	**6.02**	**12.08**		
		Enablers	**3.56**	**3.50**	**7.06**		
Evidence and evaluation	Evaluation methods	Barriers	5.22	3.67	8.89		
		Enablers	1.30	0.74	2.04		
	Findings	Barriers	3.30	3.60	6.90		
		Enablers	0.75	1.22	1.97		
	Focus of evidence	Barriers	4.42	3.10	7.52		
		Enablers	1.38	1.53	2.91		
	Future research	Barriers	4.65	1.41	6.06		
		Enablers	1.50	1.11	2.61		
	Total subthemes	Barriers	**17.59**	**11.78**	**29.37**		Barriers in upper quartile
		Enablers	**4.93**	**4.60**	**9.53**		Enablers in upper quartile
Integration of care	Concept of integration	Barriers	2.09	3.34	7.43		
		Enablers	4.21	1.76	5.97		
	Effectiveness of integrated care	Barriers	3.86	4.86	8.72		
		Enablers	2.82	3.23	6.05		
	Impact of integration	Barriers	1.40	4.06	5.54		
		Enablers	1.43	3.28	4.71		
	Total subthemes	Barriers	**7.35**	**12.26**	**19.61**		
		Enablers	**8.46**	**8.27**	**16.73**		
Professional roles	Community stakeholders	Barriers	1.54	0.85	2.39		
		Enablers	1.01	3.31	4.32		
	Employment and training	Barriers	2.51	1.78	4.29		
		Enablers	0.78	0.87	1.65		
	Leadership	Barriers	3.57	1.72	5.29		
		Enablers	3.23	1.81	5.04		
	Link worker role	Barriers	2.35	0.43	2.78		
		Enablers	2.00	0.36	2.36		
	Professional identity	Barriers	3.80	1.87	5.67		
		Enablers	1.09	0.63	1.67		
	Total subthemes	Barriers	**13.77**	**6.65**	**20.42**		
		Enablers	**8.11**	**6.98**	**15.09**		
Service user factors	Accessing care	Barriers	1.70	3.51	5.21		
		Enablers	1.58	5.54	7.12		
	Person-centered ethos	Barriers	1.50	1.64	3.14		
		Enablers	1.84	4.72	6.56		
	Service user outcomes	Barriers	0.30	2.57	3.50		
		Enablers	2.10	3.20	4.67		
	Total subthemes	Barriers	**3.50**	**7.72**	**11.22**		
		Enablers	**5.52**	**13.46**	**18.98**		
	Overall total subthemes	Barriers	**57.70**	**48.87**	**106.57**		
		Enablers	**42.30**	**51.13**	**93.43**		

The values in bold text represent total subthemes and overall total subthemes.

### 3.1 Collaborative approach

#### 3.1.1 Collaboration and partnership

The subtheme collaboration and partnership was a key enabler within both the review articles and grey literature with the highest relative frequency for the latter advocating “a strong partnership approach that works across sectors and gives attention to power and building trusting relationships with communities” as one of the five most important principles of community-centered public health [(26), p. 10]. Review articles referred to the concept of partnership synergy and the mechanisms underlying partnership functioning such as building trust in collaboration:

it is clear that maximising initial trust through any means possible is essential to starting on the right foot and for increasing the chance of rapidly achieving synergy and avoiding inertia. So, changing the context through the use of stronger legal agreements to uphold roles and responsibilities, fostering a shared vision, and drawing upon prior collaborative experience, as well as putting in place robust accountability and governance arrangements in case of conflict, can all go a long way to increasing initial trust level and mitigating further loss of trust if/when conflicts occur [(27), p. 19].

#### 3.1.2 Co-production

The grey literature showed that the subtheme co-production was a key enabler. Two review articles (28, 29) reported a growing interest in, and evidence for, the use of co-production in healthcare services:

This scoping review provides ample evidence that complex health interventions, service improvements and applied research are being co-designed and co-produced with patients, the public and other stakeholders, and supports current knowledge about the diverse processes and formats of co-production… Instead of trying to define a gold standard in co-production, we argue for accepting the diversity in approaches to co-production and call on researchers to be clearer in their reporting. Different approaches are needed to tailor co-production to context, different stakeholder groups and various stages of the research and implementation process [(29), p. 40].

Several grey literature resources stressed that working co-productively with communities was a public health priority (30–32) as exemplified by the statements “communities are a central part of the public health system and community-centered ways of working should be integral to whole system of action to improve population health”; “building healthy, resilient, connected and empowered communities is a public health priority shared across many sectors” [(32), p. 16]; and “recognizing the protective and risk factors at a community level that affect people’s health, and how these interact with wider determinants of health” [(32), p. 10]. Increased coordination of effort appeared to be the primary motivation for organizations seeking to co-operate, as explained:

Inter-organizational collaborations can take many different shapes and forms. Whether associated with terms such as partnership working, partnering, or integration, these entities have been touted to bring a range of advantages over competitive approaches by enabling innovations, improving coordination of effort (i.e., reduce duplication, improved information sharing), enabling access to greater resource, gaining greater influence over others, and strengthening relationships [(27), p. 2].

Another consideration was the voluntary nature of co-production, for example:

the coming together of local government and often myriad health organizations from the public, private and third sectors is essentially a voluntary exercise driven by what is perceived to be best for patients and the wider population – but with remarkably little ability to compel anyone to do anything [(20), p. 6].

As one of eight key messages, the author wrote “There is agreement that if this essentially voluntary approach to coordinating care better can be achieved, then it will stick, and probably more firmly and effectively than if it was merely mandated by legislation” [(20), p. 5].

#### 3.1.3 Inter-professional relationships

The subtheme of inter-professional relationships was found to be a key enabler within review articles. Seven articles indicated that professionals can actively contribute to inter-professional collaboration (33–39) as illustrated:

Although the evidence is limited and fragmented, the 64 studies in this review show professionals are observed to contribute in at least three ways: by bridging multiple types of gaps, by negotiating overlaps in roles and tasks, and by creating spaces to do so. Studies predominantly focus on physicians and nurses, and results show active albeit different efforts by both professional groups. The data provide some evidence that collaborating requires different efforts by professionals involved within either teams or network settings, as well as within different subsectors [(39), p. 339].

### 3.2 Costs

#### 3.2.1 Cost effectiveness

Within grey literature, the subtheme of cost effectiveness was a key barrier and covered the challenging financial position of some organizations (40), difficulties attracting funding or interest from social prescribing commissioners (41), and costs excluded from cost analysis (42), for example:

Traditionally cost analysis considers the costs incurred to develop and implement an intervention, including direct costs, indirect costs, and intangible costs. Direct costs represent the value of resources used specifically for the intervention. These costs are often characterized as medical or non-medical. Direct medical costs can include costs such as clinical services, diagnostic tests, medications etc. These are always included in cost analyses. What is less often included are the direct nonmedical costs such as those associated with a public health intervention. For something like social prescribing this could include things like developing a media campaign, training, materials, community of practice and peer support events, the cost of advertising etc. [(42), p. 6].

Six review articles however found insufficient evidence about the factors that determine cost-effectiveness (28, 43, 44) and three review articles found no clear evidence as to whether models of integrated care were cost neutral, or cost less or more (45–47), as exemplified by:

Although some studies have found that integrated care is associated with lower costs, others highlight that improved integrated care tends to uncover unmet needs, with a detrimental effect on cost. Furthermore, integrated care may lead to cost savings for one organization while increasing costs for another [(47), p. 54].

#### 3.2.2 Cost savings

While the subtheme of cost savings was not a key barrier, three review articles found trade-offs in sustainability and performance for scaling-up between mandated versus voluntary and small versus large collaborations, networks versus single organizations, and types of governance structures (48–50) as explained:

Trade-offs exist between being small enough to maintain flexible and inclusive decision-making processes, and being of sufficient size to influence the local health economy, bear financial risks, and meet the administrative requirements of regulation… Some evidence suggests that non-hierarchical organizations, co-operatives, and professional partnerships tend to compete for contracts on quality rather than price, as they try to maintain their members’ incomes and working conditions. In comparison, corporate provider organizations, which may not face the same type of pressures to maintain employees’ interests, may be better placed to compete for contracts on price. However, in doing so purchasers may need to rely on complex incentive schemes and short-term contracts in order to better align the corporate provider’s organizational goals with those of the NHS. Such levers may be expensive to control and maintain in the long term [(49), p. 54].

### 3.3 Evidence and evaluation

#### 3.3.1 Evaluation methods

The subtheme of evaluation methods acted as a key barrier to integrated care; seven review articles found insufficient evidence of evaluation due to heterogeneity within social enterprises, contexts within which they operated and the wide variety of health impacts (28, 34, 43, 47, 49, 51, 52), as described:

Our original research question sought to ascertain whether social enterprises provide improved outcomes in comparison with usual care in health and social care systems. Additionally we sought to understand the importance of context through distinguishing between studies according to whether the social enterprise activity occurred in a competitive or collaborative healthcare system. With reference to our original research question there is insufficient evidence to provide conclusive answers. In part this is because of the heterogeneity of organizational types included within the label social enterprise, the wide variety of health impacts that different studies have focused upon, and the variety of contexts in which these social enterprises operate [(43), p. 1806].

#### 3.3.2 Findings

Although the subtheme of findings was not a key barrier, authors of both review articles and grey literature found little research into new forms of collaboration (49), that evidence was of moderate quality (53), and was limited in determining whether social inclusion can be enhanced via green, blue, and public space interventions; community infrastructure could reduce loneliness; or whether social inclusion could be enhanced by public space interventions (31), for example:

There is strong evidence that community infrastructure is a necessary but not sufficient factor for thriving communities, as it provides a place in/at which people can meet and, in some cases, it can be a focal point of the local area. For this infrastructure to support the community effectively, it needs to be utilised for purposes that facilitate community networks and interactions (i.e., to provide opportunities for bridging, bonding and linking groups of people), often led by activity organizers or community-based institutions [(31), Executive Summary].

#### 3.3.3 Focus of evidence

The subtheme focus of evidence was found to be a key barrier by review articles. Four articles, for example, reported a disproportionate focus either on micro-level interventions with a lack of focus on meso-organizational and macro-system levels in which programs operate (54, 55) or on micro- and meso-level interventions with a lack of focus on macro-system levels (56, 57), as characterized by:

Overall, we observed a relative low proportion of organizational (meso) level and system (macro) level integration interventions (and therefore elements), compared with micro level interventions, in the included reviews. The emphasis on the micro level is consistent with findings on studies of development and implementation of models of care generally. This disproportionate micro level emphasis most likely reflects the complexity in tackling whole-of-system issues (i.e., from the micro level through to the macro level), both in terms of implementation and measurement complexity, resulting in a one-dimensional focus to integrated care interventions and their evaluation. Health and/or social care system change or re-emphasis requires targeted interventions at multiple levels – micro, meso, and macro [(54), p. 7].

Additionally, review articles found the focus of studies to be too narrow, as demonstrated by:

the majority of contributions provide recommendations related to a smaller number of specific aspects that were found to be influential. Moreover, these were often derived in specific contexts/settings or with defined target patients, especially to those with chronic illnesses as opposed to those with comorbidities or wider health and social care needs. Few studies propose, and eventually validate, frameworks indicating key areas of intervention and/or analytical aspects to consider in order to foster care integration [(58), p. 10].

#### 3.3.4 Future research

Review articles found that the subtheme was key barrier as further research was needed to explore person-centered experiences, priorities of consumers and focus on how families and carers were involved (35) and identify experiences and priorities of clinicians in inter-professional approaches (34). The following comment was made as one of a set of policy points “Policymakers should critically evaluate integrated care programs to identify and manage conflicts and tensions between a program’s aims and the context in which it is being introduced” [(59), p. 446]. Future research was covered to a lesser extent by grey literature which suggested that significant gaps in the data at the interface between health and social care at a national level needed to be addressed (60) and that, in reference to ICSs “even in areas that were frontrunners of this approach, the formal structures and mechanisms that were put in place by the legislation have yet to be fully tested” [(60), p. 7].

### 3.4 Integration of care

#### 3.4.1 Concept of integration

Within review articles, the subtheme of concept of evaluation was a key enabler though mentioned rarely within grey literature. Several review articles referred positively to one or more of the terms collaboration, co-production, coordination and co-location of services, and co-shared responsibility, for example, “Current knowledge about integrated care provides the basis for the development of integrated care initiatives, but to take integrated care a step further, deeper understanding of collaboration and behaviour in integrated care is needed” [(61), p. 2]; “An integrated care approach specifically for multi-morbidity requires that integration and coordination of care go beyond the traditional single-disease focus” [(56), p. 32]; “Where relationships were built on trust and respect, the level of care coordination was enhanced” [(35), p. 1161]; and “Fragmentation of systems and services should be avoided since this has a negative impact on the experiences of users of IHSC [integrated health and social care]. The use of intermediate IHSC services along with more effective coordination can potentially go some way to address this issue” [(35), p. 1165].

#### 3.4.2 Effectiveness of integrated care

Within grey literature, the subtheme of effectiveness of integrated care was a key barrier covering variation in ICS’s working practices:

The number of joint posts between organizations; shared commissioning practices; mutual scrutiny arrangements; the role of local politicians, and local priorities and who will lead on their implementation – though the list is extensive. This variation is the product of multiple factors including, but by no means limited to, the strength of relationships between senior leaders in systems, the extent to which joint-working arrangements were already in place pre-ICS, and the boundaries around which ICSs have been set [(60), p. 7].

Other authors observed that “regulators and national bodies have been slow to align how they work with ICSs, and this is particularly evident in the way in which regional teams of NHS England and NHS Improvement relate to NHS commissioners and providers” [(40), p. 5].

#### 3.4.3 Impact of integration

Within grey literature, the subtheme of impact of integration was a key barrier though mentioned little in review articles. High frequency barriers questioned how load bearing ICSs should become (20) and proposed that the work was not seen as the preserve of patient experience and public engagement teams alone (19). Furthermore, impact of integration was affected by issues brought about by COVID-19, such as managing recovery from the pandemic against the backdrop of a cost-of-living crisis (60) and keeping up-to-date, for example: “where a large-scale NHS structural reform is underway within a pandemic-influenced world, there is a real risk that the new resultant structures will be out of touch with communities before they even get started” [(62), p. 1].

### 3.5 Professional roles

#### 3.5.1 Community stakeholders

Grey literature showed that the subtheme of community stakeholders was an enabler with around average frequency though the topic was covered only briefly by review articles. Guides and reports observed a need to build on pre-existing local resources such as the VCSE sector (63), that recognition of contributions might not need to be financial, written acknowledgement or support for developing skills could be provided instead (64) and a whole systems’ approach would enable local communities to share an understanding of the reality of the challenge (26). Additionally, “ongoing consultation with community stakeholders and relevant partners from the health and third sectors is critical, and organizations should invest time in developing these relationships” [(41), p. 77].

#### 3.5.2 Employment and training

Within review articles, the subtheme of employment and training presented a greater barrier than an enabler though frequencies were relatively low across all literature. Barriers included “staff turnover,” “limited financial resources to fund service providers or secure a high salary for employed staff” [(65), p. 10] and “a need for improved communication between professionals and better information technology to support them, greater clarity about who is responsible and accountable for physical health care…” [(52), p. 8].

#### 3.5.3 Leadership

Three review articles showed that issues around the subtheme of leadership acted as barriers; more profound knowledge was needed to build a stronger evidence base and supportive interventions to develop leadership skills to warrant the current emphasis on leadership in integrated care (33, 37, 38), as illustrated:

the use of leadership as the implementation strategy, although recommended in the Chronic Care Model and by many experts in the field, was hardly applied or described since we only found two studies of low and mediocre quality that evaluated leadership-training interventions aimed at structurally supporting implementation processes of integrated care. This shows that the importance of leadership to integrated primary care does not yet transcend the level of opinions [(38), p. 16].

Furthermore, power and influence used by integrated service leaders and hierarchies between health and social care complicate the leading of integrated teams and systems, as typified by:

Although integrated care is designed to make care more efficient, the associated complexities that are perhaps less visible, more nuanced, or deliberately suppressed (e.g., professional hierarchies, embedded tension, unconscious biases, political motivations) encourage complex consequences across the system. The need for this to be managed, or at least kept at bay, by clarity of message from those in positions of authority is another natural response to uncertainty. The difficulty with which this is genuinely provided by leadership, who are expected to recognize these hidden, embedded complexities and offer communication that is both clear and mindful of this is, therefore, considerable [(51), p. 65].

#### 3.5.4 Link worker role

The subtheme of link worker role was covered to a greater extent by review articles than grey literature though there was little difference in frequency between barriers and enablers. Barriers included issues around services delivered by link workers rather than health care professionals as explained:

Patients may be wary about speaking to someone they do not know; how the messenger (e.g., health care professional, written information) broaches seeing a link worker as an option should be given consideration; otherwise, there is a risk of it being rejected by patients. If a link worker is serving several practices (e.g., in a primary care network), then waiting lists may increase; this could jeopardise “buy-in” from patients and health care professionals [(66), p. 13].

As enablers, review articles represented link workers as a vehicle for accruing social capital such as trust, sense of belonging and practical support to give patients confidence, motivation, connections, knowledge, and skills to manage own health and well-being thereby reducing reliance on general practitioners (65, 66) as described:

Patients may consider, with the link worker, ways of resolving potential barriers (e.g., due to travel, childcare). This enables them to move forward in life, becoming connected to community resources, so they feel less isolated and more in control of their situation. Making new connections through the link worker can result in patients no longer fixating on personal problems… we inferred that through developing “buy-in” and strong relational connections, link workers mobilize resources that come from being part of social networks. We propose that these networks then prompt patients to feel more able and willing to manage their own health goals [(66), p. 7–8].

#### 3.5.5 Professional identity

Within review articles, the subtheme of professional identity, which included professional roles and behaviour, was seen as a barrier with around average frequency. Researchers found that “Adopting an inter-professional, community-orientated and population-based primary care model requires a fundamental transformation of thinking about professional roles, relationships and responsibilities” and that “Team-based approaches can replicate existing power dynamics unless medical clinicians are willing to embrace less authoritarian leadership styles” [(37), p. 76].

### 3.6 Service user factors

#### 3.6.1 Accessing care

The subtheme of accessing care was a key enabler of integrated care within grey literature though also could be seen as a barrier. As an enabler, the subtheme identified a need for creativity within referral pathways as “some individuals may find it easier to refer themselves or avoid dealing with certain professionals for a variety of reasons (including negative past experience with health and/or social care services)” [(67), p. 39]. Statutory guidance additionally recommended improving care access for minority groups, such as:

auditing and monitoring the participation of certain groups, for example in events and formal governance roles to help identify any gaps in engagement requiring attention and to support staff to promote the involvement of people who are more reflective of the population in question [(64), Equality Considerations].

With respect to heritage site considerations, though more widely applicable, barriers compromising the promotion of inclusivity were described as “physical accessibility of properties, material barriers such as the cost of transport or tickets, and a lack of representation and training in the workforce” [(41), p. 76].

#### 3.6.2 Person-centered ethos

The subtheme of person-centered ethos was a key enabler within grey literature but also a barrier occurring with average frequency. As an enabler, there was a need to ensure that voices were heard “from all parts of the community – not just those who speak loudest or those the system has traditionally found easiest to engage with” [(63), p. 9] and to ask the right questions:

Rather than asking questions about people’s experiences of individual services, ask questions focused on partnership working, the coordination of services and people’s experience of this. How do services work together around people’s needs in a way that makes sense to them? What matters to people and what will make a difference to their lives? [(63), p. 9].

As a barrier, it was recognized that traditional ways of working involved “what is the matter” with an individual, not “what matters” to them and that there was a “need to move to a more holistic approach” [(62), p. 1].

#### 3.6.3 Service user outcomes

The subtheme of service user outcomes was seen as a barrier of just below average frequency within grey literature and only covered to a limited extent by review articles. The highest levels of barrier concerned safeguarding:

There is a responsibility to keep people who get involved safe. Being asked repeatedly to go back over bad or even traumatic experiences so professionals can learn how to improve services, will cause distress and increase lack of trust. One way to keep people safe is not to create isolated positions of “lay representation” which can burden people with the responsibility of bringing a public perspective to a large group of professionals. Instead it can be better to work with groups who can continue to support each other outside of meetings and help each other to take part effectively. Depending on the context, it may be necessary to arrange therapeutic support at activities and afterwards [(64), p. 47].

## 4 Discussion

The current rapid evidence review aimed to appraise and synthesize review articles and grey literature covering integrated care outcomes to determine barriers and enablers of integrating health and social care and community resources. Thematic analysis revealed barriers and enablers of integrated care and content analysis ascertained the extent of this evidence. References made to barriers of integrated care in review articles exceeded those of enablers by a factor of more than a third whereas they were roughly equal for grey literature. The theme, Evidence and Evaluation showed the highest frequency of barriers in review articles for subthemes evaluation methods, focus of evidence, and future research and the theme, Collaborative Approach showed the highest frequency of enablers in review articles for subthemes collaboration and partnership, and inter-professional relationships and in grey literature for subthemes collaboration and partnership, and co-production.

For Evidence and Evaluation, a key barrier was found to be the difficulty of evaluation due to the heterogeneous nature of social enterprises, their operational contexts, and the wide variety of health impacts (43). Alongside this barrier was a disproportionate focus on micro-, or micro- and meso-levels, rather than macro-level research, and on specific contexts or defined service user groups (54). Explanations for the limited scope of these approaches included issues with operationalizing and measuring wider macro-systems level research or whole systems (micro to macro) research (54). The finding of the micro-level focus on defined groups especially those with chronic illness is particularly pertinent for service users with comorbidities or wider health and social care needs (58) as the research indicates these populations have not been well-served by the integration of care. The authors drew attention to the need to accurately define client groups, profile their needs, and manage the complexity by designing care for clients with complex medical and non-medical conditions (58). Other authors reported the necessity of high-quality research and transparent reporting (15) and the importance of assessing and measuring the right aspects of integrated care (19). To allow primary care to refocus resources on prevention, the recent Hewitt Review pointed out that a new more holistic approach should recognize that “outcomes rather than just activity need to be measured” [(68), p. 66]. The current review ascertained areas needing further research were those which comprised priorities and experiences of service users and clinicians, along with a comprehensive and systematic evaluation of integrated care to identify and manage the interactions between program aims and their contexts.

For Collaborative Approach, a key enabler was the active contribution which healthcare professions could make to the organization of inter-professional relationships, such as by bridging gaps and negotiating overlaps in roles and tasks (39). Audit Scotland’s framework of features to support integration included collaborative leadership and building relationships with agreed governance and accountability arrangements, and ability and willingness to share information (15). Further enablers within Collaborative Approach occurred within the subtheme of collaboration and partnership which created interest and produced evidence (29). Outcomes mainly accounted for the diversity of approaches to co-production tailored to the contexts in which integration was taking place (29), and of partnership synergy referring to the mechanisms underlying partnership functioning such as building trust and faith in the collaboration (27). Fostering a shared vision, drawing upon previous collaborative experience, and putting in place stronger legal agreements and arrangements for robust accountability and governance were seen to maximise trust and thereby increase the achievement of partnership synergy (27).

Less clear cut in its outcomes was the theme of Integration of Care which showed barriers in the grey literature (subthemes: effectiveness of integrated care and impact of integration) but enablers in the review articles (subtheme: concept of integration). The finding implied that a positive view was taken of the theoretical concept of integration but its effectiveness and impact in practical terms indicated room for improvement (69). Integration of Care was seen as the basis for care initiatives going beyond a traditional single-disease focus to deal with multi-morbidities (56), with relationships built on trust and respect, and systems that avoid fragmentation (35), albeit with the need for deeper understanding of collaboration (61). Authors found that ICSs offered “real potential for partnership with the NHS and other sectors, with an opportunity to develop genuinely joined-up, personalized care” [(19), p. 1]. Barriers within the same theme concerned the effectiveness and impact of integrated care where a need for a better understanding of the factors that drive behaviour, decision-making, collaboration, and governance processes were seen to be needed (61). Additionally, cohesive strategies were necessary to design medical and non-medical care for specific populations (48) and policymakers needed to allow time for integration to embed as the field was regarded as still far from maturity (28). Furthermore, there was concern about unequal power relationships exemplified by the following comment that “although ICSs are intended to promote equal partnership between the NHS and its wider partners, including local authorities and social care, the history of previous attempts at integration suggests there is a risk that the NHS will dominate” [(19), p. 1].

Within the grey literature, the theme of Costs (subtheme: cost effectiveness) was a key barrier. Findings showed a lack of evidence about factors that determined cost-effectiveness, specifically whether models of integrated care cost less, more, or were cost neutral (45, 46) and trade-offs in sustainability and performance from scaling-up that might not necessarily have produced economies of scale, for example in competing for contracts (49). In keeping with the paucity of evidence about cost effectiveness and cost savings revealed by the current review, other authors postulated that integrated care needs to make better use of public money by clearly articulating evidence of its impact (19) and that current investment in the NHS was not creating the best health value that it could (68). The Audit Scotland Report framework stressed the need for integrated finances and financial planning to support integration of the service (11).

Analysis of the grey literature indicated that both Service User Factor subthemes (accessing care and person-centered ethos) had high relative frequencies as key enablers though there was considerably less coverage of these subthemes in the review articles. It is likely, therefore, that authors of grey reports, guides and other resources in the public domain were more concerned about issues from the point-of-view of the end user than of those reading peer-reviewed publications. Enablers for accessing care included creativity within the referral process such as self-referral (67); identifying gaps in participation among specific populations; targeting resources and approaches at minority groups; and using accessible venues (64). Enablers for a person-centered ethos involved participatory methods to navigate complex socio-economic challenges and strengthen the legitimacy of decision-making (70); NHS organizations involving the public (64); asking the right questions such as people’s experience of coordinated services (63) and employing creative and flexible approaches to meet the needs of individual service users (71, 72).

Also notable was the theme of Professional Roles and although no subthemes lay in the top quartile, the frequency of barriers and enablers were approximately equal suggesting that this subtheme met with the least consensus. In particular, the subtheme leadership, with similar relative frequencies for both barriers and enablers, served to highlight the current emphasis on leadership in integrated care. Leaders’ relational, organizational, and change-management skills were viewed as important to improve care integration (38). Leadership culture was regarded as critical (19) though the authors pointed out the need for clarity about structures and system. Other authors noted the lack of support from a strong evidence base for the roles of leaders and the development of leadership skills and training (38) and that there should be a focus on improving outcomes for their populations through “strengthening local leaders’ ability to have greater and more flexible decision making in primary and social care, supported through a more joined up national policy approach” [(68), p. 64]. Furthermore, leadership in integrated care appeared to be compromised by prior professional hierarchies, tensions, unconscious biases, and political motivations existing within healthcare and social care teams (51).

Given the wide-ranging outcomes of integrated care within the reviewed literature, it seems surprising that only about 2% of the coverage concerned the need to address inequalities despite it often being expressed as an aim of ICSs. The Hewitt Review, for example, stated that one of the four aims of ICSs of bringing together the main partners in a common purpose was to “tackle inequalities in outcomes, experience and access” [(68), p. 4]. Public Health England advocated a “radical shift” to “put communities at the heart of public health” and “reduce widening and persistent health inequalities” [(32), p. 4] asserting that “community-centered approaches are increasingly used in public health practice to enhance individual and community capabilities, create healthier places and reduce health inequalities” [(32), p. 5]. It is possible, however, that the “opportunity to focus on prevention, population health and health inequalities might be treated as a “nice to have” that must wait until the immediate pressures upon the NHS had been addressed and NHS performance recovers” despite the view that “prevention, population health management and tackling health inequalities are not a distraction from the immediate priorities: indeed, they are the key to sustainable solutions to those immediate performance challenges” [(68), p. 12].

### 4.1 Limitations

As there is no single definition of rapid reviews in the literature and no agreed methodology for conducting rapid reviews (22), the current rapid evidence review used several modifications to the full systematic review (often regarded as the gold standard of reviews) to produce evidence relatively quickly with the needs of decision-makers in mind whilst maintaining integrity and methodological quality. Modifications involved using targeted research objectives to determine the barriers and enablers of integrating health and social care and community resources and ascertaining the extent of this evidence; a reduced list of sources searched limited to published, peer-reviewed review articles and grey literature resources in the English language, with one reviewer for study selection and data extraction checked by a second. Furthermore, although the terms “enablers” and “barriers” are widely used, these concepts cannot be clearly demarcated in that a barrier once it is overcome may become an enabler, rather than an absolute obstacle. For the purposes of this review, however, the identification of whether outcomes were barriers and enablers was based upon how the outcomes were expressed by the authors of the reviewed resources. Due to the limitations of the research, therefore, broad consensus about the conclusions regarding barriers and enablers of integrated care should be treated relatively cautiously. The potential issue of publication bias is also acknowledged as studies appeared to report fewer positive outcomes or enablers of integrated care; consequently, these articles might have been inadvertently under-represented.

## 5 Conclusion

The current rapid evidence review of review articles and grey literature from 2018–2022, conducted thematic and content analyses to determine barriers and enablers of integrated care in the UK. The main barriers were the paucity of robust research by which to evaluate interventions involving health and social care services in partnership with community stakeholders (62, 73, 74), and a lack of standardized methods for assessing cost effectiveness (42). The current review demonstrated that providing sufficient cost evidence is an importance area of research which still needs to be fully addressed (49), in conjunction with providing clear and transparent outcomes that can be fed back to policy makers. The main enablers of integrated care were the organizational skills of health and social care professionals who were actively able to contribute to inter-professional collaborations by bridging task-related gaps and overlaps (75), and a growing interest in co-production rather than competition in healthcare services to improve information sharing and reduce duplication (76). It was noticeable, however that there appeared to be a lack of emphasis on addressing health inequalities (68). Several factors were found to be involved in bringing about integrated care which, in addition to structural integration, included coherent policies; joint strategies across organizations; and “political, managerial and clinical leadership with a clear and consistent focus on integrated care” [(12), p. 80]. It is important though to bear in mind the World Health Organization view that “any integrated model development is strongly contextually-bound, nearly impossible to replicate and can only be successful if it does account for unique needs and characteristics of the population it aims to serve” [(77), p. 1]. To these ends, it is critical to identify elements of integrated care associated with successful outcomes (11) and determine which interventions are meaningful and sustainable in the long term with the objectives of person-centred coordinated care and the wider use of preventive approaches to population health (19).

## Author contributions

LT: Conceptualization, Data curation, Formal analysis, Investigation, Methodology, Visualization, Writing – original draft, Writing – review & editing. HC: Conceptualization, Funding acquisition, Investigation, Project administration, Supervision, Validation, Writing – review & editing.
