# Supplementary material for: Barriers and enablers of integrated care in the UK: a rapid evidence review of review articles and grey literature 2018–2022
Source: Front Public Health. 2024 Jan 4;11:1286479. doi: 10.3389/fpubh.2023.1286479 (PMC10794528; doi:10.3389/fpubh.2023.1286479)
Supplement: Supplementary file 1 [file Table_1.docx]

Supplementary Table S1. Review articles: Narrative summary of outcomes.

| **Reference (no.) name** | **Review type (dates)** | **Methods**  **(no. of studies)** | **Barriers/ Enablers** | **Narrative summary of outcomes** |
| --- | --- | --- | --- | --- |
| (56) Alderwick et al. | Systematic narrative review of reviews  (2001-2019) | Review studies (36) | Barriers | - Benefits of collaboration may be hard to deliver and measure and possibly overestimated by policymakers. - Evidence of impact on health services, resource use, and spending is mixed. - Little convincing evidence to suggest that collaboration between local health care and non-health care organizations improves health outcomes. - Little known about which kinds of collaborations work, for whom, and in contexts. - Local collaborations need to be understood within their macro-level political and economic context. |
|  |  |  | Enablers | - Collaboration between local health care and non-health care organizations might contribute to better population health. - Studies spanned collaborations with broad population health goals (preventing disease and reducing health inequalities) to those with a narrower focus (better integration between health care and social services). |
| (27)  Aunger et al. | Realist review  (1990-2020) | Case studies, reviews, case-control studies, theoretical articles (53) | Barriers | - If too many conflicts occur, and too many tasks are not achieved, trust in collaboration can be broken leading to dissolution and lack of faith in collaboration. - Increased integrative or mandated collaboration may lean on contracts to drive collaborative behavior. - Lack of information exchange between partners as needed leads to lack of coordination. - Program theories incorporated concept of “collaborative inertia”. |
|  |  |  | Enablers | - Degree to which organizations are coordinated is key to underlying success or failure of collaborations. - Essential to consider mechanisms underlying partnership functioning, such as building trust and faith in collaboration, to maximize synergy and thus collaborative performance. - Increased coordination is a primary motivation for organizations seeking to cooperate; leads to reduction in duplication of effort and gaps in services and sharing of knowledge and skills. - Program theories incorporated concept of “partnership synergy” - mechanisms underlying partnership functioning such as building trust and faith in collaboration. |
| (48)  Baxter et al. | Systematic review  (2006-2017) | Systematic reviews; RCTs; non-RCTs; longitudinal and cross-sectional studies (167) | Barriers | - Contradictory outcomes, studies showed both increased and decreased use of community services. - No clear evidence as to whether costs are neutral, increase or reduce. |
|  |  |  | Enablers | - Improved access to care services. - Increased patient satisfaction. - Perceived improved quality of care by staff. - Reduced outpatient appointments and waiting time. |
| (49)  Baxter et al. | Systematic review  (2006-2017) | Systematic reviews; RCTs; non-RCTs; observational and qualitative studies (267) | Barriers | - More staff time may be required. - No clear evidence as to whether costs are neutral, less or more. |
|  |  |  | Enablers | - Improved access to care services. - Increased patient satisfaction. - Perceived improved quality of care by staff. - Potential for patients with complex needs. - Reduced outpatient appointments and waiting time. |
| (33)  Best, Williams | Scoping literature review  (1980-2018) | Peer-reviewed journals (16) | Barriers | - Fears of integrated working. - Roles of others can challenge development of professional identity of practitioners in interprofessional teams. - Social care staff more likely to apply for management posts than health professionals. Implications for leadership and management (such as the “reluctant manager”). - Uni-professional training can give narrow or distorted understanding of another’s roles. |
|  |  |  | Enablers | - Mitigated by two management strategies: i) effective planning; ii) reporting benefits back to staff to ensure they retain confidence in the change. - Trust is vital component when negotiating professional identity during a period of change. |
| (54)  Briggs et al. | Review of reviews using systematic search  (2005-2016) | Systematic reviews of RCTs; reviews of RCTs and non-RCTs (15) | Barriers | - Disproportionate focus at micro level and relative lack of information regarding the meso-organizational and macro system-level care integration strategies compared with micro level interventions. - Elements of integrated care for older people focus particularly on micro clinical care integration processes. |
|  |  |  | Enablers | Key elements of existing models include multidisciplinary team care, comprehensive assessment, and case management. |
| (34)  Bunn et al. | Realist synthesis  (2004-2017) | Primary research: RCTs, quantitative and qualitative studies; reviews; guidelines; case studies; reports; opinion pieces (88) | Barriers | - Service providers need support to change professional behavior and better organize and deliver services. - To embed shared decision-making in practice requires radical shift from biomedical focus to a more person-centered ethos. |
|  |  |  | Enablers | - Face to face interactions, permission, space to discuss options, and continuity of patient professional relationships are key. - Future research needs to focus on inter-professional approaches and how families and carers are involved. - Four mechanisms need to be in place i) understanding and assessing patient and carer values and their capacity to access and use care, ii) organizing systems to support and prioritize decision-making, iii) supporting and preparing patients and family carers to engage in shared decision-making, and iv) a person-centered culture of which decision-making is part. - Programs likely to be successful are those that allow older people to feel respected and understood, and that engender confidence to engage. |
| (43)  Calò et al. | Systematic literature review  (1994-2017) | Primary research; RCTs, quasi-experimental, case control and case studies (25) | Barriers | - Insufficient evidence to provide conclusive answers partly due to heterogeneity within label of social enterprise, the wide variety of health impacts that different studies focused upon, and the variety of contexts in which social enterprises operate. - Social enterprises had higher costs than not-for-profit organizations, but lower costs than public sector. |
|  |  |  | Enablers | - Positive results in quality of life, activities daily living score and changing behavior. - Possible improved physical health and mobility and improved mental health, though not depression. - Where provision via a social enterprise has positive health outcomes, a collaborative environment appears to facilitate benefits to wellbeing and mental health. |
| (50)  Coates et al. | Scoping review  (2003-2018) | Quantitative and qualitative studies; and review articles (43) | Barriers | - Future research is needed to identify elements of integrated care associated with outcomes, measure cost implications and identify experiences and priorities of consumers and clinicians. - No clear evidence as to whether models of integrated care are cost neutral, increase, or reduce cost. - Studies low in evaluation. |
|  |  |  | Enablers | - Found 37 models of integrated care with key characteristics: shared information technology, financial integration, single-entry point, multidisciplinary teams and meetings, co-located, coordinated, and person-centered care, agreed referral criteria, joint assessment, treatment plan and treatment. - Increased service access and improved physical and mental health outcomes. - Models well received by consumers and providers. |
| (55)  Dambha-Miller et al. | Scoping review  (1996-2020) | Empirical research: grey literature; newsletters; discussion papers; reports, blogs; webinars and working papers (84) | Barriers | - Focus on individual level services rather than multi-level or multi-sector integration of services for which there is limited evidence. - Improving clinical care in one or two sectors may not be as effective as simultaneously improving the organization or design across services as one single system of provision. - Inherent tension between top-down and bottom-up approaches to integrated care. - May take time to establish single system of provision and will require local input. - Policymakers need to allow time for integration to embed, to enable new structures and relationships to develop and mature. - Wider determinants of population health likely to require integration beyond primary care and social services. |
|  |  |  | Enablers | - Increasing emphasis on the need to consider broader determinants of population health. - Requires a whole-systems structure while allowing for local flexibilities. |
| (44) Farmanova et al. | Scoping review  (2000-2017) | Published articles, reports and grey literature (57) | Barriers | - Need expanded assessments to evaluate patient needs for social and medical care to better understand effectiveness of multifaceted interventions. - Need to find creative ways of addressing clinical and non-clinical issues (housing surgeries in primary care). - Need to focus on health and wellness addressing intersectoral action and partnerships, health in vulnerable groups, and wide range of determinants of health. - Need vast social and provider networks to support patients with complex needs and broaden scope of services. |
|  |  |  | Enablers | - Benefits for access to care and services such as same day appointments. - Combining integration of care with population health approach requires cohesive strategies to design medical and non-medical care for defined populations and redesign of service organization and delivery. - Higher level of perceived care quality. - Improvements in morbidity and mortality rates and overall care quality. - Increased uptake of screening and immunization. - Reductions in waiting time for referral, emergency room use, hospital admissions, 30-day re-admissions and length of stay. - Some initiatives show potential for cost-containment, cost-savings, and reduction in care cost. |
| (28) Fusco et al. | Bibliometric analysis  (1994-2019) | Academic journal articles and reviews (295) | Barriers | - Field is still far from maturity. - Practices, especially regarding co-delivery and co-management as well as the evaluation of their real impacts on providers and on patients are lacking and should be more widely investigated. |
|  |  |  | Enablers | - Fast-growing interest in co-production in healthcare. - Strong commitment to co-production by the UK government and the NHS to cut costs and improve efficiency of public services. |
| (58) González-Ortiz et al. | Literature review  (2006-2016) | Peer-reviewed and grey literature articles (18) | Barriers | - Development of conceptual frameworks needed to understand and guide thinking on integrated care which has evolved over time. - Most reviewed studies make recommendations related to a small number of influential aspects often derived in specific contexts or with defined target patients especially those with chronic illness, as opposed to comorbidities or wider health and social care needs. |
|  |  |  | Enablers | - Range of elements and factors associated with successful care integration. |
| (52)  Harris et al. | Realist synthesis  (1994-2019) | Empirical studies and grey literature (36) | Barriers | - Often focused on personality of leader of an integrated team or service rather than their specific role. - Paucity of evidence and empirical research of patient perspective. - Power and influence used by integrated service leaders and hierarchies between health and social care identified which complicate the leading of integrated teams and systems. |
|  |  |  | Enablers | - Evidence identified for seven potentially important components of leadership in integrated care teams and systems: inspiring intent to work together; creating the conditions to work together; balancing multiple perspectives; working with power, taking a wider view; commitment to learning and development; and clarifying complexity. |
| (35) Henderson et al. | Integrative literature review  (2007-2018) | Primary research in peer-reviewed journals including quantitative, quantitative,  and mixed methods (20) | Barriers | - Lack of clarity and difficulty in understanding and navigating integrated systems. - Need for further research that explores person-centered experiences. - People who access integrated health and social care services feel that they are not always involved in planning their care subsequently impacting on their experiences of the services. - Service user and informal carer voices appear under-represented in current literature and studies that included their views are of low quality overall. |
|  |  |  | Enablers | - International evidence that effective collaboration can be facilitated through co-location of services that enhance professionals’ understanding of each other's roles. - Relationships grow stronger when providers approach care planning with involvement and collaboration. - Relationships hold significance, and potential impact of relationships between professionals and service users should not be underestimated. - UK staff highlighted that inter-professional communication is as important to collaborative working, regardless of co-location. |
| (59)  Hughes et al. | Systematic hermeneutic review  (1999-2018) | Empirical, studies; policy literature; grey literature; evidence reviews (71) | Barriers | - Integrated care is not a unified concept but an emergent set of practices. - Need to critically evaluate integrated care to identify and manage tensions between program aims and context. - Inability of professionals to integrate. - Patient subjective experiences may not match objective organizational achievements. - Patients unable to exercise full control over their care. |
|  |  |  | Enablers | - Association between improved patient experiences and system benefits. - Person-centered, relationship-based care can potentially contribute to (but not determine) improved patient experiences. |
| (73)  Kelly et al. | Systematic review of reviews  (1998-2018) | Systematic and non-systematic literature reviews (18) | Barriers | - Challenges to measuring integration identified of wide range of potential impacts. - Difficulties in comparing findings due to differences in study design, and heterogeneity of outcomes. - Integrated care needs to be comprehensively and systematically evaluated if it is to be implemented widely. - Large number of available outcome measures and infrequent use of any core measurement sets make comparisons between schemes difficult. |
|  |  |  | Enablers | - Increased recognition of need for services to bring together a range of professionals and skills from across the health and social care sector. - Integration of care is intended to benefit the service user. |
| (69) Kozlowska et al. | Narrative literature review  (2000-2017) | Original research articles; one review and one discussion article (14) | Barriers | - Inadequate payment mechanisms between organizations. - Insufficient focus on patient needs and wishes when planning and delivering care. - Lack of commitment by organizations involved, conflicting organizational interests, and insufficient resources to develop an integrated service. - Misunderstandings over care priorities. - Moving care to primary care without upskilling workforce. - Poor exchange of patient information between healthcare professionals. - Poor coordination of care. - Resistance to change. - Tensions between healthcare professionals due to uncertainty over new roles and responsibilities. |
|  |  |  | Enablers | - Requires synchronized changes on different levels: well-resourced team equipped with additional finance, time, and team members; well-defined, evidence-based service; agreed and articulated new roles and responsibilities; willingness among healthcare professionals to co-work and co-learn; general practices working together; shared goals and values across organizations; improved electronic communication; fostering commitment and enthusiasm for joint working; and monitoring care quality and performance. |
| (36)  Lawless et al. | Scoping review  (2008-2017) | Empirical, interpretative, and critical research reporting quantitative, qualitative, and mixed methods and grey literature (3) | Barriers | - Conflicting information, clinical advice, treatments and/or management. - Lack of opportunity to clarify patient needs and priorities including those related to patient information and communication; need arrangements that reflect patient preferences about which services to access, speed and methods of access with appropriate user-friendly technologies. - Lengthy wait times. - Limited interprofessional or multidisciplinary teamwork and relational and informational discontinuity at the primary–secondary care interface. - Unavailability of providers and services in certain areas. |
|  |  |  | Enablers | - Need clear communication coupled with suitable information and systems to reduce information gaps and enable regular follow-up. - Need individualized care planning with appropriate patient involvement in decision-making, and regular contact with familiar and trusted healthcare provider. |
| (74)  Liljas et al. | Systematic review and narrative synthesis  (1995-2018) | RCTs, quasi-experimental designs; comparison studies; survey evaluation (12) | Barriers | - Effectiveness of integrated care on patient outcomes in later life largely unknown. - Further theory-based research is needed to establish the effect of integrated care on patient-related outcomes. - Lack of robust findings. - No significant changes in mortality rate. |
|  |  |  | Enablers | - Integrated care tends to reduce hospital admission rates and length of stay, and possibly readmission and patient satisfaction. - None of studies reported integrated care having a negative impact on outcomes assessed. |
| (37)  Miller et al. | Scoping review  (2007-2017) | Peer-reviewed primary research (10) | Barriers | - Engagement of patients and communities often limited due to lack of capacity and lack of belief it will make an impact. - Internal (relationships, cultures, experience of improvement) and external (incentives, policy intentions, community pressure) contexts can derail transformation efforts. - Team-based approaches can replicate existing power dynamics unless medical clinicians are willing to embrace less authoritarian leadership styles. |
|  |  |  | Enablers | - Adopting inter-professional, community-oriented, and population-based primary care model requires fundamental transformation of thinking about professional roles, relationships, and responsibilities. - Internal (relationships, cultures, experience of improvement) and external (incentives, policy intentions, community pressure) contexts can encourage transformation efforts. - Transformation requires coordinated programs that incorporate external facilitation of change; learning through training and reflection; and formative and summative evaluation. - Transformation requires developing clinical and non-clinical leaders, engaging community and professional stakeholders and transitional funding. |
| (38)  Nieuwboer et al. | Systematic literature review  (2006-2018) | Empirical studies situated in an integrated primary care setting; mapping of qualitative data on leadership skills (20) | Barriers | - Good quality research on clinical leadership in integrated primary care is scarce. - Little support that leaders positively influence implementation of integrated care. - More profound knowledge needed about leadership skills and integrated-care implementation, and leadership support aimed at developing these skills. - Research to build stronger evidence base for leadership and supportive leadership interventions is needed to warrant current emphasis on leadership in integrated primary care. |
|  |  |  | Enablers | - Leaders’ relational and organizational skills as well as process-management and change-management skills considered important to improve care integration. - Physicians seemed to be the most adequate leaders. - Two non-controlled studies suggest that leadership support programs helped prepare and guide leaders and positively contributed to implementation of integrated primary care. |
| (65)  Pescheny et al. | Narrative synthesis  (2011-2016) | Evaluation reports and one conference report (8) | Barriers | - Collaborative approach to project management results in lack of targeted approach to strategic and robust project management; absence of robust risk management systems; volunteers as navigators; staff turnover; limited financial resources to fund service providers or secure high salary for employed staff; lack of shared understanding among stakeholders and partners. - General practice staff and patient disengagement. - Lack of partnership and service level agreements. - Need more high-quality research and transparent reporting of findings. - Need phased roll out implementation approach and realistic planning of “lead in time” to set up service rather than “go live dates” approach to implementation. - Reduction in available and suitable service providers in the third sector. |
|  |  |  | Enablers | - Need link worker-ready general practices with general practice staff engagement and a culture that supports biopsychosocial model of health. - Need flexibility during development, implementation, and delivery. - Need good relationships and effective communication between stakeholders within and across sectors with shared understanding, attitudes, and perspectives of stakeholders. - Need social prescribing champions in clinical commissioning groups and general practices. - Need wide range of good quality third sector service providers. - Need workshops to design and discuss service prior to implementation and standardized training, briefings, and networking events for partners. |
| (45) Pettigrew et al. | Narrative synthesis  (1996-2016) | Peer-reviewed and grey literature (46) | Barriers | - Economies of scale from larger organizations may not always outweigh diseconomies of scale which may emerge due to new more complex governance and management processes. - Little research into new forms of collaboration. - Should not be assumed that integration of services is a straightforward intervention which will improve cost effectiveness or save money but instead as range of complex interventions aiming to achieve long-term changes in way health services are delivered. - Since the impact and potential unintended consequences are not yet clear, it would be advisable for policymakers to move with caution and be informed by ongoing evaluation. - Trade-offs exist in “scaling-up” between mandated and voluntary collaboration; networks versus single organizations; small versus large collaborations; and different types of governance structures in terms of sustainability and performance. - While positive impact seems plausible, evidence suggests it is not a given that clinical outcomes or patient experience will improve, nor that cost savings will be achieved by increasing organizational size. |
|  |  |  | Enablers | - Concept of integrated care described as “polymorphous” in nature. - Engagement of GPs is essential to increase the likelihood of collaborations succeeding; GPs must feel they have sufficient autonomy and influence over any new groupings. |
| (46)  Rocks et al. | Systematic literature review  (2001-2019) | RCTs; cohort and cross- sectional studies (34) | Barriers | - Evidence varies largely and is of moderate quality, insufficient evidence about factors that determine cost-effectiveness of integrated care. |
|  |  |  | Enablers | - Significant decrease in costs and increase in patient outcomes compared with non-integrated controls (care as usual) especially in studies with follow-up of over a year. |
| (53)  Rodgers et al. | Mapping review  (2013-2017) | Systematic and non-systematic reviews; primary studies; book chapters; grey literature (45) | Barriers | - Efforts to improve physical health care of people with severe mental illness should empower staff and service users and help to remove everyday barriers to delivering and accessing integrated care. - Lack of continuity of care and lack of integration across service settings. - Service models described inadequately, and few evaluated. - Need to improve staff communication, offer more technological support, provide greater clarity about who is responsible and accountable for physical health care, and greater awareness of effects of stigmatization. |
|  |  |  | Enablers | - Care coordinators may have an empowerment role in providing advocacy for service users and might benefit from greater formal authority over care integration. |
| (72)  Sadler et al. | Systematic review and narrative synthesis  (2009-2017) | Qualitative and mixed methods studies (18) | Barriers | - Complexity of care needs of patient population, difficulties with system navigation and access, and limited service user and carer involvement in care decisions. - Similarities and differences in lay and professional stakeholder perspectives on integrated care for older people with frailty. - System level factors influencing implementation largely reported by providers included limited support for service users and carers to navigate and access health and care system, limited involvement of service users and carers in care decisions, unavailability of infrastructure to support and fund integrated care; and limited staff capacity and training. |
|  |  |  | Enablers | - Multi-dimensional ways in which integrated care was understood from perspectives of service users, carers and providers. - Providers emphasized improved care coordination of between providers in different sectors. - Service users and carers highlighted continuity of care with professional they could trust. |
| (39)  Schot et al. | Systematic review  (2010-2020) | Empirical peer-reviewed qualitative and mixed methods studies (64) | Barriers | - Differences exist between collaborative settings and healthcare subsectors. - Professionals from different professions seem to make different contributions. |
|  |  |  | Enablers | - Evidence for professionals actively contributing to interprofessional collaboration in three distinct ways: by bridging professional, social, physical, and task-related gaps (more likely to be nurses than physicians), by negotiating overlaps in roles and tasks, and by creating spaces/organizing. |
| (51)  Shahzad et al. | Scoping review  (1990-2017) | Empirical studies (45) | Barriers | - Further research is needed to develop outcome measures for successful collaboration and determine which interventions are sustainable in the long term. |
|  |  |  | Enablers | - By linking clinical services across primary care with those of other sectors such as public health, healthcare practices can enhance patient follow-up and improve health outcomes, reduce duplication of services, and achieve economies of scale. - Findings highlight interventions that address inequities in care by targeting underserved, high-risk population groups. |
| (29)  Smith et al. | Scoping review  (2018-2020) | Empirical  studies; reviews; guidelines; opinion pieces or commentaries (83) | Barriers | - Co-production needs capacity to implement co-produced interventions to become more embedded in organizational structures. - No studies have critically reflected on how co-production in applied health research might evolve. |
|  |  |  | Enablers | - Ample evidence that complex health interventions, service improvements and applied research are being co-designed and co-produced with patients, public and other stakeholders. - Interest in and use of co-production in healthcare services and research is growing. - Skill set, multiple levels of engagement, negotiation, funding, and institutional arrangements needed for meaningful co-production. - Support for current knowledge about diverse processes and formats of co-production. |
| (47)  Soley-Bori et al. | Systematic review  (2004-2021) | Original research articles and grey literature (17) | Barriers | - Depression is main cost-increasing condition. - Issues of data measurement due to variability in social economic status in small geographic areas. - Multimorbidity increases healthcare costs and utilization of primary and secondary care with large negative effect on unplanned hospitalization. |
|  |  |  | Enablers | - Country-specific focus aims to better inform UK healthcare policy. - Provides evidence in support of NHS England’s policy of expansion of integrated care schemes. |
| (57) Struckmann et al. | Scoping review  (1996-2015) | Quantitative and qualitative research on models and elements (92) and programs (50) | Barriers | - Gaps in scientific evidence, for example on financing mechanisms, that could strengthen care integration and control or save cost if programs are to be sustainable and widely adopted. - Models mostly focus on micro- and meso-level context and do not discuss macro environment in which programs operate |
|  |  |  | Enablers | - Key elements comprised person-centered care, holistic assessment, self-management, integration and coordination of services, and collaboration. |
| (66)  Tierney et al. | Realist review  (2018-2019) | Empirical studies and grey literature (118) plus information obtained from CCGs through Freedom of Information Act | Barriers | - Ending patient contact with link worker needs to be given consideration from the onset. - How the messenger (healthcare professional, written information) broaches seeing a link worker is important otherwise risk of link worker being rejected by patients. - If link worker is serving several practices in a primary care network, then waiting lists could increase and could jeopardize “buy-in” from patients and healthcare professionals. - Patients may be wary about speaking to someone they do not know. - Unintended consequences such as increased demand on GPs. |
|  |  |  | Enablers | - Developing wider social networks prevents people from feeling isolated and exposes them to alternative perspectives and experiences of the world, giving life meaning. - Link workers represent vehicle for accruing social capital (trust, sense of belonging, practical support) which gives patients confidence, motivation, connections, knowledge, and skills to manage own health and well-being, thereby reducing the burden on GPs. |
| (61) Zonneveld et al | Systematic review  (2006-2017) | Empirical, theoretical, and conceptual articles (22) | Barriers | - Although substantial generic knowledge about integrated care has been developed, better understanding of factors that drive behavior, decision-making, collaboration, and governance processes is needed. - Articles mostly written from academic perspective so difficult to draw conclusions about professional or service user views. |
|  |  |  | Enablers | - Frequently identified values are: “collaborative”, “coordinated”, “transparent”, “empowering”, “comprehensive”, “co-produced”, and “shared responsibility and accountability”. - Values provide better understanding of behaviors and collaboration in integrated care. |
