# Supplementary material for: Barriers and enablers of integrated care in the UK: a rapid evidence review of review articles and grey literature 2018–2022
Source: Front Public Health. 2024 Jan 4;11:1286479. doi: 10.3389/fpubh.2023.1286479 (PMC10794528; doi:10.3389/fpubh.2023.1286479)
Supplement: Supplementary file 2 [file Table_2.docx]

Supplementary Table S2. Grey literature: Narrative summary of outcomes.

| **Reference (no.) name** | **Resource type (dates)** | **Method (no. of Ps/studies)** | **Barriers/ Enablers** | **Narrative summary of outcomes** |
| --- | --- | --- | --- | --- |
| (76) Agastin et al. | Report  (2009-2022) | Survey of senior ICS staff (301) by Department of Health and Social Care and NHS England | Barriers | - Challenges faced by NHS and care providers exacerbated by pandemic. - Challenging nature of financial savings targets. - High risk of lack of clarity of objectives, insufficient resources, and unclear governance and accountability. - Inherent tension between meeting national targets and addressing local needs. - Health and care providers face longstanding financial and operational challenges such as high level of staff vacancies in NHS and social care. - ICSs may be too focused on health services. - Need time and capacity to build relationships and design services that better meet local needs. - NHS focus on current performance rather than longer term population health management. - Some aspects of ICSs are still in their infancy or being developed. |
|  |  |  | Enablers | - Better integration of health and care services can help to remove barriers to collaboration, reduce duplication, create economies that increase cost-effectiveness, and improve patient experience. - Functional integration of resources needed, for example, sharing information and digital assets. - Joint planning and delivery of services can reduce gaps or overlaps in provision and bring related or complementary services together. - NHS England consulted extensively in designing ICSs and widely supported by majority of stakeholders. - Pooling resources and budgets across geographical boundaries or service types to meet shared aims. - Reduction of patient contact points to services ensures continuity of care and data accessible to all partners. |
| (71) Bryer et al. | Evaluation  (2016-2021) | Desk-based research; meta-analysis of funded projects (77); fieldwork with subset of projects (15); survey of ICF project leads (68); and interviews and focus groups with stakeholders (74) from all RPBs (7) | Barriers | - Annual nature of funding creates uncertainty for projects. - Effects of pandemic. - Staff recruitment and retention. |
|  |  |  | Enablers | - Adequate resources to provide intensive and tailored support over a long period of time to individuals. - Adopting single point of contact for a project so all partner organizations can refer to it in consistent manner. - Creative and flexible approaches, that could be piloted and refined to meet needs of individual service users. - Developing resources, such as training materials, that could be used on wider level post funding. - Effective implementation of cohesive pathways of care for service users. - Enthusiastic and committed staff who work well together, learn from each other, and form cohesive, team approach around service user. - Excellent communication and effective collaboration in place between staff from across different services. - Multi agency working with shared commitment to achieving same outcomes across services. - Services becoming well established and well-known over time. - Staff who are experts in their field, highly experienced and specialists, who can effectively support services users with complex needs staff having the time and resources to support individuals in tailored way. - Well understood processes for contacting different services and drawing upon their input. |
| (40) Charles et al. | Report  (Jan-Aug 2018) | Interviews with senior NHS and local government leaders and other local stakeholders (72) in 8 of 10 first wave ICSs | Barriers | - Challenging financial position of some organizations made it harder for local systems to take collective responsibility for resources due to concerns about sharing financial risk with organizations in deficit. - Continuing tensions between statutory framework focused on organizations, their roles and accountabilities, and growing emphasis on systems and partnership working. - ICSs vary widely in size in terms of populations and number of organizations involved. - ICSs differ in terms of complexity and extent of joint working that existed prior to ICSs being established. - Leaders face competing demands on their time and priorities and sometimes struggle to devote time to ICS. - NHS architecture based on organizations working relatively autonomously rather than collaboratively. - No single ICS model, each area is developing differently according to local circumstances. - Regulators and national bodies slow to align how they work with ICSs particularly in way that regional teams of NHS England and NHS Improvement relate to NHS commissioners and providers. - Some ICSs enjoy relative overall stability, but others experience financial and/or performance challenges. |
|  |  |  | Enablers | - Agreeing shared vision and purpose early and inclusively is prerequisite for effective system working. - Continuity of leadership and longstanding and respected leaders identified as key enablers of change. - ICSs report working more collaboratively to manage finances and performance across the system. - ICSs that are further ahead often have history of positive working relationships strengthened by regular face-to-face meetings and focused development. - Leaders in ICSs have adapted behaviors to lead using a facilitative and enabling approach. - Track record of successful service improvement can act as catalyst for further change. |
| (62) Commun-ities Leadership Group | Report  (July 2021) | Consultation with Harnessing the Power of Communities Leadership Group | Barriers | - GPs and clinicians are time poor. - Lack of connection between primary care networks and VCSE sector. - In a rapidly changing environment with large-scale NHS structural reform within a pandemic-influenced world, risk that resultant structures will be out of touch with communities. - Lack of health professional understanding of reach of voluntary sector and its connectivity to communities. - NHS feels over complicated to navigate and heavy on bureaucracy. - PCNs developed at pace risk being implemented without understanding community health and care needs. - Power imbalance between communities and NHS - Traditional ways of working involve looking at “what is the matter” with an individual, not “what matters” to them; need to move to a more holistic approach. |
|  |  |  | Enablers | - Access to social connection through local community groups or peer mentoring provides a more sustainable way of tackling issues that lead people to multiple visits to GPs and A&E. - Community power can enhance the engagement of those communities least likely to access mainstream health services and boost trust and confidence. - Harnessing power of communities and recognizing and building on existing community assets would bring greater connection and trust and help identify and deliver local solutions to tackling health inequalities. |
| (11) Convention of Scottish Local Authorities | Report  (May 2018-Feb 2019) | Summary of proposals for ensuring success of integration by group of senior officers (7) and larger group of senior stakeholders | Barriers | - Carers and representatives of service users need better support to enable full involvement in integration. - Collaboration between partners and with third and independent sectors needs improvement. - Effective working relationships with carers, service users and local communities need to be improved. - Framework for community-based health and social care integrated services needs to be developed. - Health Boards, Local Authorities and IJBs need joint understanding of respective financial positions. - Improved strategic planning and commissioning arrangements need to be put in place. - IJBs need to give clear directions to Health Boards and Local Authorities. - IJBs should be empowered to use their resources to better meet local population needs. - Partnerships to initiate or continue necessary conversations to make integration work, be clear about risks and ensure mitigation of risks in place. - Understanding of accountabilities and responsibilities between statutory partners needs to be improved. |
|  |  |  | Enablers | - Ability and willingness to share information. - Collaborative leadership and building relationships with focus on shared and collaborative practice. - Identification and implementation of good practice systematically undertaken by all partnerships. - Meaningful and sustained engagement. - Monitoring and evaluation progress in achieving proposals. - Partnerships need to be innovative in progressing integration. |
| (7) Fuller | Report (Nov 2021-May 2022) | Desk-based research and case studies | Barriers | - Measure of success will be whether ICSs have meaningfully improved outcomes for groups often not well-served by traditional models. - More focus needs to be given to development and support of clinical directors beyond current arrangements provided through national contract, including local provision of sufficient protected time to meet the leadership challenges in integrated neighborhood teams. - Need to ensure a particular focus on unwarranted variation in access, experience and outcomes. - Primary care networks need to make routine use of population data to inform design of care. - Recruitment and retention challenges evident across wider primary care workforce. |
|  |  |  | Enablers | - Ensuring that integrated primary care models can adapt their offer is vital in improving health outcomes and reducing future demands on the health service. - ICSs have role to play in developing a more coherent approach to digital transformation in primary care that focuses on improving patient experience. |
| (67) Fullham et al. | Handbook  (2003-2021) | Desk-based research and interviews and workshops with stakeholders including GPs, patient/user representatives, providers, social prescribing link workers, and VCSE representatives | Barriers | - Individuals that do not traditionally access intervention may be part of other community groups or networks. - Interventions may incur cost for participants, for example, outdoor clothing that could be provided for free. - Labels and language particularly around mental health may have negative connotations or stigma attached. - Lack of evidence for long term effects of intervention; effects can dissipate relatively rapidly. - Loss of social support when intervention comes to an end can be challenging for participants. - Proximity to participants, public transport links, and public access all have bearing on whether participants continue post-intervention; participants need help to identify places they can access. |
|  |  |  | Enablers | - Being creative with referral pathways, individuals may find it easier to refer themselves or avoid dealing with professionals for various reasons including negative past experiences with health/social care services. - Co-creation of interventions and programs with end users. - Link workers need to identify and create links with individuals who do not traditionally access interventions. - Signposting to local voluntary groups provides continuity for participants from activity and social perspective. - Skills acquired should have some universality in daily life (for example meditation, mindfulness, journaling, team building, leadership skills) to be applied to everyday stressors, situations, and relationships. - Successful intervention supports lasting change or enduring benefit for participants. |
| (30) Ganga et al. | Review  (2013-2022) | Systematic search and narrative synthesis of peer-reviewed journal articles and grey literature (14) | Barriers | - Barriers to cultural participation include cost, timings, location, and lack of information. - Social context can act as a barrier to participation. |
|  |  |  | Enablers | - Arts-based approach helps to break down barriers, fosters appreciation, and increases engagement. - Community-settings can influence individual and group experiences and offer potential for change and improving wellbeing and social inequalities. - Flexible delivery allows different levels of engagement for different lengths of time. - Informal and safe learning environment. - Long-term partnerships established between communities and university. - Place-based narratives facilitates connections between people, objects, and events in urban landscape. - Sufficient duration and resources ensure sustainability and self-empowerment as long-term legacies. - Social context can facilitate participation. |
| (31) Gov.UK | Review  (2001-2022) | Rapid evidence review of peer-reviewed journal articles and grey literature (115) | Barriers | - Communities differ significantly. - Limitations and gaps in robust evaluation of community initiatives including how they were selected, what local need they met, how financially sustainable they were and what would have happened without them. - Limited evidence that community infrastructure can reduce loneliness by being a point of contact, creating paid and volunteering opportunities, and encouraging social interaction. - Limited evidence as to how higher levels of social capital relate to improved lifestyle in terms of health. - Limited evidence that social inclusion can be enhanced via green, blue, and public space interventions, community organizations and community-led social enterprises. - Limited evidence that social prescribers can remove barriers that stop patients from participating. |
|  |  |  | Enablers | - Local context is important as there is no one-size-fits-all. - Medium evidence that effective community infrastructure and social capital can lead to positive social outcomes including reduced loneliness, homelessness, offending rates, and pressure on frontline services. - Medium evidence that sense of belonging, ownership and pride can be enhanced by stronger relationships, neighborhood design and public gathering spaces, associated with higher levels of wellbeing. - Medium evidence that social cohesion can be enhanced via funding schemes for grassroots community groups, community hubs and social enterprises. - Strong evidence that community infrastructure, strong social connectedness and social capital can have positive impact on community resilience and increase wellbeing. |
| (41) The Heritage Alliance | Report  (2017-2020) | Summary of case studies (31) | Barriers | - Difficulties involved in undertaking research in the cultural sector. - Organizations should explore existing resources to consider what fits their project; no one-size-fits-all. - Organizations experienced difficulties in attracting funding or interest from social prescribing commissioners and needed to find alternative funding sources. - People from socio-economically disadvantaged groups and Black, Asian, and minority ethnic communities tend to be under-represented in cultural activities. - Promotion of inclusivity compromised by physical accessibility of properties, cost of transport or tickets, and lack of representation and training in workforce. - Qualitative evidence needs combination of semi-structured interviews or focus groups, participant observation and retrospective assessment. |
|  |  |  | Enablers | - Engaging with intended audience from outset shapes project to suit their needs appropriately. - Evaluation needs to be built in from start, invest time in considering program aims to determine right evaluation methodology to make robust case to funding bodies. - Focus on senior-level buy-in to ensure project develops with cross-sector support. - Ongoing consultation with community stakeholders and relevant partners from health and third sectors is critical; organizations should invest time in developing these relationships. - Partnerships across sectors and with universities to share resources and expertise are key to success. - Partnerships leverage areas of expertise, skills, and understanding and often have a greater impact. - Share existing expertise through targeted training for other practitioners wanting to work in this space. - Working with local community, third sector and voluntary organizations can be a route to engaging with people who might benefit from interventions. |
| (60) Impower | Report  (Sept-Oct 2022) | Desk-based research, surveys, interviews (34) with representatives from local authorities (7); and roundtables with ICS participants (3) | Barriers | - Existing metrics tend to have narrow focus on outcomes and expenditure that reflect silos of services rather than wider complex system and overarching outcomes for people as they move between those services. - Formal structures and mechanisms put in place by legislation have yet to be fully tested. - ICSs at early stage of development even in areas that were frontrunners. - Intense demands in managing recovery from Covid against backdrop of cost-of-living crisis, rising demand across services, profound workforce challenges and funding restraints. - Many national datasets are inconsistent, published data on length of hospital stay of low quality, and no national data on NHS spending out of hospital. - Primacy of place but little focus on place in national policy. - Significant gaps in data at interface between health and social care at national level. - Trade-offs between treatment and preventative activity well established in health policymaking and optimism that ICSs will help to move balance between two toward more preventative activity. - Variation in ways that ICSs work include number of joint posts between organizations. shared commissioning practices, mutual scrutiny arrangements, local priorities and who leads on implementation. |
|  |  |  | Enablers | - Importance of collaborative working between health and social care services highlighted by pandemic. - Local government and NHS broadly in agreement on overall aims of ICSs as set out by central government. |
| (9) Integrated Care Fund | Annual report  (2018-2019) | First ICF report: National level overview of investment by RPBs and insight into funded projects and services and their impacts | Barriers | - Whilst individual projects currently demonstrate benefits and impacts of their individual services, difficult to describe this at program or national level due to wide variation of projects funded. |
|  |  |  | Enablers | - Evaluating impact and outcomes. - Focus on both direct and indirect support for carers. - Improving communications in relation to program delivery and impacts. - Mainstreaming of learning and new models of delivery. - Prevention and early intervention. - Regionalization and integration. - Social value sector investment and delivery. - To support integration agenda, Welsh Government made education sector, local authority housing and housing associations statutory members of RPBs. |
| (10) Integrated Care Fund | Annual report  (2019-2020) | Second ICF report: National level overview of investment by RPBs and snapshot of funded projects and services | Barriers | - Minister for Health and Social Services brought partners together to reflect on what is working, what can be done to improve shared learning and challenges around mainstreaming. - More robust reporting arrangements with emphasis on demonstrating impact of projects on people. - Review of governance around ICF to ensure appropriate scrutiny arrangements in place for decisions made by RPBs. |
|  |  |  | Enablers | - Mapping out all funding streams across health and social care to ensure better alignment of funding and aid partners to take more strategic approach to deploying collective resources. |
| (42) Kimberlee et al. | Evidence briefing  (2000-2022) | Systematic search of online databases for studies, reports or evaluations meeting criteria (34) | Barriers | - Decision makers would benefit by knowing financial resources required to implement each effective intervention and how money invested in an intervention compares to outcomes achieved. - Direct non-medical costs less often included in cost analysis including costs associated with public health intervention such as developing media campaign, advertising, training, materials, and peer support events. - Economic analyses need to go beyond quality-adjusted life-years (QALY) to take account of all stakeholders and not just the NHS or health system. - Economic evaluations comparing costs and consequences of public health interventions with existing interventions usually require randomized controlled trial (RCT) approach; researchers argue that social prescribing initiatives are not suitable for RCT approaches to cost effectiveness: - Public health decision makers faced with limited resources must routinely make decisions about how to prioritize public health problems and choose among alternative interventions. - Results remain inconclusive despite comparatively rich and deep data.   VCSEs in England report inadequate reimbursement and are forced to support costs through own budgets. |
|  |  |  | Enablers | - Consistent approach needed to collect range of data, including financial and economic costs, personal impacts, delivery models, demographics and outcomes relating to social determinants of health. - Cost analysis to consider costs incurred to develop and implement intervention including direct costs, indirect costs, and intangible costs |
| (64) NHS England and Department of Health and Social Care | Statutory guidance  (2006-2022) | Research in partnership with organizations with experience of working with communities, input from NHS England’s networks and forums, and case studies | Barriers | - Deprivation and poverty can prevent people taking part in activities unless financial support and resources in place that enable involvement. - Health literacy levels and language should be relatable rather than using NHS terms and acronyms. - People at different levels of understanding about NHS structures. - Public involvement should not be left until last moment without enough time to carry out fair and proportionate exercise when public could and should have been involved earlier or to a greater extent. - Responsibility to keep people involved safe; being asked repeatedly to go back over bad experiences so professionals can learn how to improve services causes distress and increases lack of trust. |
|  |  |  | Enablers | - Asking people what form of recognition they would value and would support them to take part. - Auditing and monitoring participation of certain groups to help identify gaps in engagement requiring attention and support staff to promote involvement of people more representative of population. - Building people’s knowledge of how different parts of health and care system fit together and where their input will make a difference. - Building relationships based on trust, particularly with marginalized groups. - Consistent approach to recruiting, training, and supporting range of people in different roles helps ensure knowledge, skills, and confidence to contribute effectively. - Good practice for organizations to have written policy giving details about reimbursing and paying people. - Making sustainable use of NHS resources. - NHS organizations need to involve public alongside duties to act effectively, efficiently, and economically. - Payments to support participation from more diverse groups and help recognize value that organizations place on people contributing their experiences, knowledge, and skills. - Practical considerations to ensure different people can take part by making activities accessible. - Recognition of cost to partner organizations in terms of staff time to provide insight, take part in advisory groups and arrange for communities to participate in engagement activities. - Recognition of people’s contributions beyond financial means including receiving acknowledgement in writing, support to develop skills and experience, and seeing improvements made because of their input. - Targeting resources and approaches at ethnic minority groups to provide additional support that some members will need because of health disparities they experience. - Using accessible venues, making reasonable adjustments or specific effort to ensure disabled people, autistic people and people with a learning disability can participate. |
| (75) Pett et al. | Annual report  (Sept 2021-Feb 2022) | Desk-based research, responses (50) to survey from ICSs (35) sent to all ICSs (42) and interviews with health and care stakeholders | Barriers | - No national NHS workforce strategy since 2003. - Risk that if ICS leaders are not given sufficient time and space, they will not be able to deliver the radical changes to health and care services that the pandemic has demonstrated are needed. - Staffing gaps that have developed and have become entrenched across health and care partly explain why recent staff absences due to COVID-19 have been so detrimental to NHS performance. - Uncertainty about how experiences and insights of those leading primary care service at neighborhood level inform system-level planning and strategy |
|  |  |  | Enablers | - Further NHS support needed to help systems achieve. - Improved joint working between partner organizations catalyzed by the pandemic. - System leaders in social care sector are innovating to plug gaps in government support. |
| (70) Pollard et al. | Report  (Feb 2021) | Roundtables of network and case studies (10) | Barriers | - Community approaches and initiatives required to demonstrate own worth according to measures not set up to recognize their value; value best captured qualitatively yet metrics are quantitative. - Community approaches are pluralistic, often small-scale and rooted in local context, but policymakers seek uniform and scalable approaches. - Community focus on long-term impact, but short- term financial and political priorities drive system. |
|  |  |  | Enablers | - Communities have wealth of knowledge and assets within themselves. - Community approaches demonstrate that cohesion is most sustainably built from the ground up. - Investing in community approaches generates greater impact for existing spend and saves money longer-term. - Involving people in decision making, alongside supporting them with resources and wider social infrastructure, can enable community action to improve wellbeing and resilience locally. - More power and resources given to local communities rather than held by central government or public services. - Participatory methods navigate complex socio-economic challenges and strengthen legitimacy of decision-making. - People need to be active participants in all efforts to improve their health and wellbeing. |
| (26) Public Health England | Evidence briefing  (2010-2020) | Desk-based review, interviews with public health leaders from local areas (12), survey of PHE People’s Panel members (342) and roundtable with representatives (23) from local and national bodies | Barriers | - None reported. |
|  |  |  | Enablers | - Bold leadership to adapt radical approaches to reduce health inequalities. - Collaborative approach needed to tackle public health issues. - Collective bravery for risk-taking action and strong partnership approach that works across sectors and gives attention to building trusting community relationships. - Communities are a central part of public health system; community-centered ways of working should be integral to whole system action to improve population health. - Co-production of solutions with communities, based on conversations with people about health and place. - Recognizing the protective and risk factors at a community level that affect people’s health, and how these interact with wider determinants of health. - Shifting mindsets and redesigning the system, aligned to building healthy, resilient, active, and inclusive communities - Whole systems approach responds to complexity through ongoing, dynamic, and flexible way of working that enables local communities and other stakeholders to share an understanding of reality of challenge. - Undertaking insight work with communities especially those seldom heard. |
| (19) Thorstensen-Woll, Bottery | Practical guide  (Dec 2021 | Work with ICSs and roundtable held with Home Instead UK stakeholders working in and with social care sector | Barriers | - Differences in language. - Differences in leadership style. - Differences in metrics culture; NHS focus on backlog reduction. - Differences in spending power and resources. - Need for clarity about structures and systems. - Resources are a key challenge so making best use of them is essential. |
|  |  |  | Enablers | - Creating opportunity to rebalance partnership between NHS and local authorities with both parties having equal influence on decision-making. - Important to assess and measure the right things. - Opportunities for social care commissioners and providers. - Opportunity to develop genuinely joined-up, personalized care. - Potential for developing meaningful, cross-sector strategies on issues such as workforce. - Potential for learning between sectors. - Potential for NHS partnership and social care stakeholders with other sectors. |
| (63) Thorstensen-Woll et al. | Guide  (2020-2021) | Literature review, stakeholder interviews with representatives from NHS, local government and experts in patient and user experience, engagement with ICSs (4); and workshops (3) | Barriers | - Need to ask the right questions; rather than asking about people’s experiences of individual services, ask questions focused on partnership working and coordination of services, and people’s experience of these. - Work cannot be seen as preserve of patient experience and public engagement teams and should be fundamental to all areas work across ICSs, place-based partnerships, and organizations within them. |
|  |  |  | Enablers | - Data drawn from user experience placed on an equal footing with operational data at systems’ level. - Right skills, capacities, and resources may need to be brought together from across systems. - Ensuring that those with greatest needs and poorest health outcomes are engaged with and listened to, not just those who speak the loudest. - Leaders need to create the right culture in meetings and communications with staff. - Local partners need to come together to develop coordinated services focused on needs of communities. - Need clear and shared understanding among local communities about what integrated care is. - Need to build on resources that exist locally including VCSE sector organizations, patient-participation groups and patient leaders already engaging with people and communities over wide range of issues. - Voice and experience of people and communities need to be at heart of health or care organization. |
| (20) Timmins | Report  (2014-2019) | Interviews (16) with leads and chairs of ICSs and Sustainability and Transformation Partnerships (STPs) | Barriers | - Governance of STPs remains in state of flux. - Growing issue about how load bearing ICSs should become and whether they should take on responsibility for quality and financial performance as opposed to planning and implementing transformation of care. - Variation in who the chairs and leads of STPs feel they are accountable to. - Worries about the pipeline of future leaders of ICSs. |
|  |  |  | Enablers | - Agreement that if this essentially voluntary approach to coordinating care can be achieved, then it will stick, and probably more firmly and effectively than if mandated by legislation. - Relationships between NHS and local authorities appears to be improving, with population health and its determinants featuring more strongly. - Widespread agreement on skills needed to achieve progress. |
