# Supplementary material for: Barriers and enablers of integrated care in the UK: a rapid evidence review of review articles and grey literature 2018–2022
Source: Front Public Health. 2024 Jan 4;11:1286479. doi: 10.3389/fpubh.2023.1286479 (PMC10794528; doi:10.3389/fpubh.2023.1286479)
Supplement: Supplementary file 3 [file Table_3.docx]

Supplementary Table S3. Review articles: Relative frequency of barriers/enablers for themes and subthemes.

| **Themes** | **Subthemes** | **Barriers/ Enablers** | **Narrative summary of outcomes [relative frequency as a percentage]** |
| --- | --- | --- | --- |
| Collaborative approach [21.15%] | Collaboration and partnership [8.47%] | Barriers [3.99%] | - Trust in collaboration can be broken if too many conflicts occur and tasks are not achieved [0.70%] - Lack of targeted approach to strategic project management within collaboration [0.46%] - Lack of shared understanding among stakeholders and partners [0.44%] - Lack of commitment by organizations involved; conflicting organizational interests and resistance to change [0.43%] - Increased integrative or mandated collaboration may lean on contracts to drive collaborative behaviour [0.41%] - Absence of robust risk management systems [0.40%] - Differences between collaborative settings and healthcare subsectors [0.29%] - Staff turnover as result of collaboration [0.28%] - Inadequate payment mechanisms between organizations [0.20%] - Lack of partnership and service level agreements [0.19%] - Program theories incorporated concept of “collaborative inertia” [0.19%] |
|  |  | Enablers  [4.48%] | - Program theories incorporated concept of “partnership synergy” - mechanisms underlying partnership functioning such as building trust and faith in collaboration [0.92%] - Increased coordination, referring to reduction in duplication of effort and sharing of knowledge and skills, is the primary motivation for organizations seeking to cooperate [0.85%] - Collaborative environment appears to facilitate benefits to wellbeing and mental health where provision is via social enterprise [0.76%] - Collaboration between local health care and non-health care organizations [0.50%] - Degree to which organizations are coordinated is key to underlying success and failure of collaborations [0.41%] - Transformation requires coordinated programs that incorporate external facilitation of change [0.40%] - Agreed and articulated new roles and responsibilities within collaboration [0.27%] - Shared goals and values across organizations [0.22%] - Improved electronic communication [0.15%] |
|  | Co-production [5.19%] | Barriers [2.77%] | - Improving clinical care in one or two sectors may not be as effective as simultaneously improving organization across services as one single system of provision [0.72%] - For co-production to become more embedded in organizational structures, it needs capacity to implement co-produced interventions [0.64%] - Conflicting information concerning clinical advice, treatments and/or management of conditions [0.50%] - Providers and services unavailable in certain areas [0.33%] - May take time to establish single system of provision [0.29%] - Single system of provision will require local input [0.29%] |
|  |  | Enablers [2.42%] | - Growing interest in use of co-production in healthcare services, and ample evidence that complex health interventions and service improvements are co-designed and co-produced with patients, public and other stakeholders [0.96%] - Increased recognition of need for services to bring together range of professionals and skills from across health and social care sectors [0.60%] - Skill set, multiple levels of engagement, negotiation, funding and institutional arrangements needed for meaningful co-production [0.51%] - Support for current knowledge about diverse processes and formats of co-production [0.35%] |
|  | Inter-professional relationships [7.49%] | Barriers [2.67%] | - Poor exchange of patient and other information between partners leading to lack of coordination and misunderstandings over care priorities [0.72%] - Inability of professionals to integrate; needs willingness among healthcare professionals to co-work and co-learn particularly in general practices [0.68%] - Limited interprofessional or multidisciplinary teamwork at primary-secondary care interface [0.39%] - Internal contexts (relationships, cultures, experience of improvement) and external contexts (incentives, policy intentions, community pressure) can derail transformation efforts [0.37%] - Relational and informational discontinuity at the primary-secondary care interface [0.32%] - Poor coordination of care [0.19%] |
|  |  | Enablers [4.82%] | - Professionals can actively contribute to inter-professional collaboration by bridging professional, social, physical and task-related gaps (more likely to be nurses than physicians), negotiating overlaps in roles and tasks, and creating spaces and organizing [1.28%] - Shared understanding, attitudes and perspectives, and good relationships and effective communication between stakeholders within and across sectors [0.75%] - Significance and potential impact of relationships between professionals and service users [0.72%] - Enhancing professionals’ understanding of each other's roles facilitated through co-location of services [0.66%] - UK staff highlighted that inter-professional communication is important to collaborative working regardless of co-location [0.64%] - Relationships grow stronger when providers approach care planning with involvement and collaboration [0.42%] - Internal contexts (relationships, cultures, experience of improvement) and external contexts (incentives, policy intentions, community pressure) can encourage transformation efforts [0.35%] |
| Costs [9.62%] | Cost effectiveness [3.66%] | Barriers [2.42%] | - Insufficient evidence about factors that determine cost-effectiveness and no clear evidence as to whether models of integrated care are cost neutral, or cost less or more [1.47%] - Multimorbidity increases healthcare costs and utilization of primary and secondary care with large negative effect of unplanned hospitalization [0.56%] - Social enterprises had higher costs than not-for-profit organizations [0.22%] - Depression is main cost-increasing condition [0.17%] |
|  |  | Enablers [1.14%] | - Strong commitment to co-production by UK government and NHS to cut costs and improve efficiency of public services [0.42%] - Requires well-resourced team equipped with additional finance, time and team members [0.34%] - Social enterprises had lower costs than public sector [0.20%] - Transformation requires transitional funding [0.18%] |
|  | Cost savings  [6.06%] | Barriers [3.64%] | - Trade-offs in scaling-up between mandated versus voluntary, and small versus large collaborations; networks versus single organizations; and types of governance structures in terms of sustainability and performance [0.93%] - Economies of scale from larger organizations may not always outweigh diseconomies of scale which may emerge due to new more complex governance and management processes [0.70%] - Gaps in evidence on financing mechanisms which if addressed could strengthen care integration and control or save cost if programs are to become sustainable and widely adopted [0.60%] - Should not be assumed that integration of services is a straightforward intervention which will improve cost effectiveness or save money [0.57%] - It is not a given that cost savings will be achieved as a result of increasing organizational size [0.49%] - Need to broaden scope of services resulting in potential reduction in utilization and cost of care [0.35%] |
|  |  | Enablers [2.42%] | - By linking clinical services across primary care with those of other sectors such as public health, healthcare practices can reduce duplication, and achieve economies of scale [0.78%] - Significant decrease in costs and increase in patient outcomes compared with non-integrated controls (care as usual) in studies with follow-up of over a year [0.70%] - Strong commitment to co-production by UK government and NHS to cut costs and improve efficiency of services [0.50%] - Some initiatives demonstrated potential for cost-savings cost-containment, and reduction of cost of care [0.44%] |
| Evidence and evaluation [22.52%] | Evaluation methods [6.52%] | Barriers [5.22%] | - Insufficient evidence due to heterogeneity within social enterprises and contexts in which they operate, and wide variety of health impacts [1.18%] - Need expanded assessments to evaluate patient needs for social and medical care to better understand effectiveness of multifaceted interventions [0.72%] - Large number of available outcome measures and infrequent use of any core measurement sets make comparisons between schemes difficult [0.70%] - Articles mostly written from academic perspective so difficult to draw conclusions about professional or service user views [0.52%] - Difficulties in comparing findings due to differences in - study design and heterogeneity of outcomes [0.45%] - Issues of data measurement due to variability in social economic status in small geographic areas [0.44%] - Practices regarding evaluation of real impacts on providers and on patients are lacking [0.43%] - Challenges to measuring integration identified wide range of potential impacts [0.39%] - Service models described inadequately, and few evaluated [0.25%] - Studies low in evaluation [0.14%] |
|  |  | Enablers [1.30%] | - Interest in and use of co-production in healthcare research is growing [0.52%] - Ample evidence that applied research is being co-designed and co-produced with patients, the public and other stakeholders [0.27%] - Formative and summative evaluation [0.27%] - Well-defined and evidence-based service [0.24%] |
|  | Findings [4.05%] | Barriers [3.30%] | - Little research into new forms of collaboration; no studies have critically reflected on how co-production in applied health research might evolve [0.81%] - Lack of robust findings with limited amount of evidence which varies largely, has number of gaps and is of moderate quality [0.75%] - Good quality research on clinical leadership in integrated primary care is scarce [0.48%] - Contradictory outcomes on increased or decreased use of community services [0.42%] - Conflicting evidence regarding length of waiting times [0.30%] - Paucity of empirical research from patient perspective [0.30%] - No significant changes in mortality rate [0.24%] |
|  |  | Enablers [0.75%] | - Perceived improved quality of care by staff [0.42%] - Association between improved patient experiences and system benefits [0.33%] |
|  | Focus of evidence [5.80%] | Barriers [4.42%] | - Disproportionate focus either on micro-level interventions with a lack of focus on meso-organizational and macro system levels in which programs operate or on micro- and meso-level interventions with a lack of focus on macro system levels [1.08%] - Recommendations related to small number of aspects often derived in specific contexts or with defined target patients, as opposed to comorbidities or wider health and social care needs [0.97%] - Since impact and potential unintended consequences are not yet clear, policymakers should move with caution, and be informed by ongoing evaluation [0.59%] - Focus on individual level services rather than on multi- level or multi-sector integration of services [0.56%] - Interventions that address inequities in care, target underserved and high-risk populations groups [0.43%] - Elements of integrated care for older people focus on micro clinical care integration processes [0.40%] - Evidence often focused on personality of leader of an integrated team or service rather than specific role [0.39%] |
|  |  | Enablers [1.38%] | - Studies spanned collaborations with broad population health goals (preventing disease and reducing health inequalities) to those with narrower focus (better integration between health care and social services) [0.70%] - Local collaborations need to be understood within their macro-level political and economic context [0.29%] - Increasing emphasis on need to consider broader determinants of population health [0.25%] - Synchronised change needed on different levels [0.14%] |
|  | Future research [6.15%] | Barriers [4.65%] | - Need to explore person-centered experiences, priorities of consumers and focus on how families and carers are involved [0.80%] - Need to identify experiences and priorities of clinicians in inter-professional approaches [0.62%] - Need to critically evaluate integrated care to identify and manage tensions between program aims and context [0.53%] - Integrated care needs to be comprehensively and systematically evaluated if it is to be implemented widely [0.51%] - Need to identify elements of integrated care associated with outcomes [0.44%] - Need to determine which interventions are sustainable in long term [0.42%] - Practices regarding co-delivery and co-management should be more widely investigated [0.37%] - Need to develop outcome measures for successful collaboration [0.32%] - Need more high-quality research and transparent reporting of findings [0.32%] - Need to measure cost implications [0.29%] |
|  |  | Enablers [1.50%] | - Focus on health and wellness addressing intersectoral action and partnerships, health in vulnerable groups, and a wide range of determinants of health [0.60%] - Finding creative ways of addressing clinical and non-clinical issues such as housing surgeries in primary care [0.43%] - Emphasis on broader determinants of population health 0.32% - Monitoring of care quality and performance (0.15%) |
| Integration of care [15.81%] | Concept of integration [6.30%] | Barriers [2.09%] | - Integrated care should be viewed as range of complex interventions aiming to achieve long-term changes in the way health services are delivered [0.64%] - Integrated care is not a unified concept, but an emergent set of practices described as “polymorphous” in nature [0.64%] - Development of conceptual frameworks to understand and guide thinking on integrated care has evolved over time [0.56%] - Complexity of care needs of patient population [0.25%] |
|  |  | Enablers [4.21%] | - Collaboration, co-production, coordination, and co-location of services with co-shared responsibility [1.02%] - Comprehensive joint holistic care assessment and joint treatment plan and treatment [0.81%] - Person-centered care and case self-management [0.45%] - Multi-disciplinary teams and meetings [0.40%] - Financial integration, accountability and transparency [0.30%] - Country-specific focus aims to better inform UK healthcare policy [0.27%] - Agreed referral criteria [0.22%] - Shared information technology [0.20%] - Single-entry point [0.20%] |
|  | Effectiveness of integrated care [6.68%] | Barriers [3.86%] | - Need better understanding of factors that drive behavior, decision-making, collaboration and governance processes in integrated care [0.86%] - Combining integration of care with population health approach requires set of cohesive strategies to design medical and non-medical care for defined populations and to redesign service organization and delivery [0.82%] - Policymakers need to allow time for integration to embed, to enable new structures and relationships to develop and mature; field is still far from maturity [0.63%] - Inherent tension between top-down and bottom-up approaches to integrated care requires a whole-systems structure while allowing for local flexibilities [0.59%] - Wider determinants of population health are likely to require integration beyond primary care and social services [0.44%] - Effectiveness of integrated care on patient outcomes in later life remains largely unknown [0.36%] - ‘Go live dates’ approach to implementation [0.16%] |
|  |  | Enablers [2.82%] | - Integration mitigated by management strategies of effective planning, and reporting benefits back to staff to ensure they retain confidence in the change [0.61%] - Face to face interactions, permission and space to discuss options, and continuity of patient-professional relationships are key [0.48%] - Phased roll out implementation approach with realistic planning of lead in time to set up service [0.45%] - Flexibility during development, implementation and delivery [0.32%] - Standardized training, briefings and networking events for partners [0.30%] - Workshops to design and discuss service prior to implementation [0.28%] - General practice culture that supports biopsychosocial model of health [0.28%] - Substantial generic knowledge about integrated care has been developed [0.10] |
|  | Impact of integration [2.83%] | Barriers [1.40%] | - While positive impact seems plausible, evidence suggests it is not a given that clinical outcomes or patient experience will improve [0.52%] - Lack of continuity of care and lack of integration across service settings [0.30%] - Unintended consequences of integration such as - increased demand on GPs [0.28%] - Insufficient resources to develop integrated service [0.20%] - Fears of integrated working [0.10%] |
|  |  | Enablers [1.43%] | - Tends to reduce hospital admission rates and length of stay, and possibly re-admission [0.50%] - Provides evidence in support of NHS England’s policy of expansion of integrated care schemes [0.38%] - Providers emphasized improved care coordination between providers in different sectors [0.36%] - Reduced outpatient appointments [0.19%] |
| Professional roles [21.88%] | Community stakeholders [2.55%] | Barriers [1.54%] | - Collaborative approach can result in reduction of available and suitable third sector service providers [0.51%] - Limited financial resources to fund service providers [0.43%] - Need vast social and provider networks to support patients with complex needs [0.31%] - Contradictory outcomes: increased or decreased use of community services [0.29%] |
|  |  | Enablers [1.01%] | - Social prescribing champions in clinical commissioning groups and general practices [0.37%] - Wide range of good quality third sector service providers [0.27%] - Transformation requires engaging community stakeholders [0.23%] - Models well received by community providers [0.14%] |
|  | Employment and training [3.29%] | Barriers [2.51%] | - Limited financial resources to secure high salary for employed staff [0.42%] - Staff need greater clarity about who is responsible and accountable for physical health care [0.37%] - General practice staff disengagement [0.33%] - Social care staff more likely to apply for management posts than health professionals [0.32%] - Collaborative approach to project management can result in staff turnover [0.24%] - Need greater awareness of effects of stigmatization of staff [0.22%] - Moving care to primary care without upskilling workforce [0.20%] - Staff need more technological support [0.19%] - Need to improve staff communication [0.12%] - More staff time may be required [0.10%] |
|  |  | Enablers [0.78%] | - Learning through training and reflection [0.23%] - Engaging professional stakeholders [0.21%] - Fostering commitment and enthusiasm for joint working [0.20%] - General practice staff engagement [0.14%] |
|  | Leadership [6.80%] | Barriers [3.57%] | - More knowledge needed to build stronger evidence base for leadership and supportive leadership interventions aimed at developing leadership skills to warrant current emphasis on leadership in integrated care [1.17%] - Power and influence used by integrated service leaders and hierarchies between health and social care complicate leading of integrated teams and systems [1.08%] - Little evidence leaders positively influence implementation of integrated care; implications for leadership (such as the “reluctant manager”) [0.66%] - Transformation requires clinical and non-clinical leaders [0.66%] |
|  |  | Enablers [3.23%] | - Leadership support programs can prepare and guide leaders and positively contribute to implementation of integrated primary care [0.74%] - Leaders’ relational and organizational skills as well as process- and change-management skills important to improve care integration [0.66%] - Leaders inspiring intent and creating conditions to work together [0.44%] - Leaders balancing multiple perspectives and taking wider view [0.40%] - Leaders’ commitment to learning and development [0.37%] - Physicians seemed the most adequate leaders [0.22%] - Leaders working with power [0.20%] - Leaders clarifying complexity [0.20%] |
|  | Link worker role [4.35%] | Barriers [2.35%] | - If link worker is serving several practices in primary care network, then waiting lists could jeopardize buy-in from patients and healthcare professionals [0.72%] - The way healthcare professional or written information broaches patient seeing link worker could risk link worker being rejected by patients [0.70%] - Ending patient contact with link worker needs to be given consideration from onset [0.34%] - Collaborative approach to project management can result in volunteers as navigators [0.33%] - Patients may be wary about speaking to someone they do not know [0.26%] |
|  |  | Enablers [2.00%] | - Link workers represent vehicle for accruing social capital such as trust, sense of belonging and practical support which give patients confidence, motivation, connections, knowledge, and skills to manage own health and well-being, reducing reliance on GPs [1.17%] - Care coordinators may have an empowerment role in providing advocacy for service users and might benefit from greater formal authority over care integration [0.66%] - Need link worker-ready general practices [0.17%] |
|  | Professional identity [4.89%] | Barriers [3.80%] | - Adopting inter-professional, community-orientated, and population-based primary care model requires fundamental transformation of thinking about professional roles, relationships and responsibilities [0.84%] - Team-based approaches can replicate existing power dynamics unless medical clinicians are willing to embrace less authoritarian leadership styles [0.58%] - Health care service providers will need support to change their professional behavior and to better organize and deliver services [0.47%] - Evidence often focused on personality of integrated team or service leader rather than their specific role [0.44%] - Roles of others can challenge development of professional identity of practitioners in inter-professional teams [0.43%] - Tensions between healthcare professionals due to uncertainty over new roles and responsibilities [0.39%] - Uni-professional training can give narrow or distorted understanding of another’s roles [0.34%] - Professionals from different professions seem to make different contributions [0.31%] |
|  |  | Enablers [1.09%] | - Trust is a vital component when negotiating professional identity during a period of change [0.39%] - Engagement of GPs is essential to increase the likelihood of collaborations succeeding [0.37%] - GPs must feel they have sufficient autonomy and influence over any new groupings [0.33%] |
| Service user factors [9.02%] | Accessing care [3.28%] | Barriers [1.70%] | - People who access integrated health and social care feel they are not always involved in planning their care and unable to exercise full control over their care leading to limited involvement in care decisions and subsequent impact on experience of services [0.72%] - Patient difficulties with understanding, accessing and navigating integrated systems; need to remove everyday barriers to accessing care [0.70%] - Efforts to improve the physical health care of people with severe mental illness should empower staff and service users, and help to remove everyday barriers to delivering integrated care [0.28%] |
|  |  | Enablers [1.58%] | - Arrangements that reflect patient needs and preferences about which care services to access, and improved and quicker access to these services [0.56%] - Clear communication coupled with suitable information, such as user-friendly technologies, with systems to reduce information gaps and enable regular follow-up [0.36%] - Reductions in emergency room use, hospital admissions, 30-day re-admissions, and length of stay [0.25%] - Individualized care planning with appropriate patient involvement in decision-making [0.15%] - Regular contact with familiar and trusted healthcare provider [0.15%] - Increased uptake of screening and immunization [0.11%] |
|  | Person-centered ethos [3.34%] | Barriers [1.50%] | - Lack of opportunity to clarify patient needs, priorities and preferences including planning and delivering care and patient information and communication [0.48%] - Limited engagement or disengagement of patients and communities due to lack of capacity and lack of belief that it will make an impact [0.43%] - Service user and informal carer voices appear under-represented in current literature; studies including their views tended to be of low quality overall [0.32%] - To embed shared decision-making in practice requires a radical shift from a biomedical focus to a more person-centered ethos [0.27%] |
|  |  | Enablers [1.84%] | - Preparing patients and carers to engage in shared decision-making; systems to support and prioritize decision-making and a person-centered culture in which decision-making is part [0.62%] - Key elements comprise person-centered care, holistic assessment, self-management, integration and coordination of services, and collaboration [0.28%] - Programs likely to be successful are those that allow older people to feel respected and understood, and that engender confidence to engage [0.28%] - Face to face interactions, permission and space to discuss options, and continuity of patient-professional relationships are key [0.25%] - Person-centered, relationship-based care can potentially contribute to (but not determine) improved patient experiences [0.21%] - Mechanisms to understand and assess patient and carer values and their capacity to access and use care (0.20%) |
|  | Service user outcomes [2.40%] | Barriers [0.30%] | - Lack of improvement in mental health for depression [0.15] - Subjective experiences of patients may not match objective achievements of organizations [0.15%] |
|  |  | Enablers [2.10%] | - Integrated care is intended to benefit the service user; developing wider social networks prevents people from feeling isolated and exposes them to alternative perspectives and experiences [0.45%] - Higher level of overall quality of care and perceived quality of care with Increased patient satisfaction and models well received by service users [0.34%] - Increased access to care and services, such as same day appointments, with reductions in waiting time for referrals [0.32%] - By linking clinical services across primary care with other sectors such as public health, healthcare practices can enhance patient follow-up and improve health outcomes [0.30%] - Some improvements in physical health outcomes in terms of mobility, morbidity and mortality rates, and mental health outcomes (though not for depression) [0.28%] - Positive results in terms of quality of life, activities daily living and changing behavior measures [0.16%] - Service users and carers highlighted continuity of care with professional they could trust [0.15%] - Some potential for patients with complex needs [0.10%] |
