# Supplementary material for: Barriers and enablers of integrated care in the UK: a rapid evidence review of review articles and grey literature 2018–2022
Source: Front Public Health. 2024 Jan 4;11:1286479. doi: 10.3389/fpubh.2023.1286479 (PMC10794528; doi:10.3389/fpubh.2023.1286479)
Supplement: Supplementary file 4 [file Table_4.docx]

Supplementary Table S4. Grey literature: Relative frequency of barriers/enablers for themes and subthemes.

| **Themes** | **Subthemes** | **Barriers/ Enablers** | **Narrative summary of outcomes [relative frequency as a percentage]** |
| --- | --- | --- | --- |
| Collaborative approach  [18.76%] | Collaboration and partnership [10.70%] | Barriers [2.89%] | - High risk of lack of clarity of objectives, insufficient resources, and unclear governance and accountability [1.03%] - Continuing tensions between statutory framework focused on organizations, their roles and accountabilities, and growing emphasis on systems and partnership working [0.76%] - Collaboration between partners and with third and independent sectors needs improvement [0.47%] - NHS feels over complicated to navigate and heavy on bureaucracy [0.36%] - Need for clarity about structures and systems [0.27%] |
|  |  | Enablers  [7.81%] | - Collective bravery for risk-taking action and a strong partnership approach that works across sectors and gives attention to power and building trusting relationships with communities [0.60%] - Minister for Health and Social Services brought partners together to reflect on what is working, what can be done to improve shared learning and challenges around mainstreaming [0.58%] - Partnerships to initiate or continue necessary conversations to make integration work, be clear about risks and ensure mitigation of risks in place [0.48%] - Joint planning and delivery of services can reduce gaps or overlaps in provision and bring related or complementary services together [0.44%] - Working with regional partners to improve ability to report on impacts and outcomes of ICF program at national level [0.39%] - Reduction of patient contact points to services ensures continuity of care and data accessible to all partners [0.36%] - Excellent communication and effective collaboration in place between staff from across different services [0.35%] - Organizations should explore existing resources to consider what fits their project; no one-size-fits-all [0.35%] - Agreeing shared vision and purpose, early and inclusively is prerequisite for effective system working [0.33%] - Partnerships across sectors and with universities to share resources and expertise are key to success [0.33%] - Partnerships leverage areas of expertise, skills, and understanding and often have a greater impact [0.33%] - Importance of collaborative working between health and social care services highlighted by pandemic [0.33%] - Identification and implementation of good practice systematically undertaken by all partnerships [0.32%] - Sufficient duration and resources ensure sustainability and self-empowerment as long-term legacies [0.32%] - Need time and capacity to build relationships and design services that better meet local needs [0.31%] - Multi-agency working with shared commitment to achieving same outcomes across services [0.29%] - Track record of successful service improvement can act as a catalyst for further change services [0.29%] - Improved joint working between partner organizations catalyzed by pandemic [0.26%] - Potential for NHS partnership and social care stakeholders with other sectors [0.26%] - Collaborative approach needed to tackle public health issues [0.20%] - Partnerships need to be innovative in progressing integration [0.20%] - Services becoming well established and well-known over time [0.20%] - Mainstreaming of learning and new models of delivery [0.17%] - Meaningful and sustained engagement [0.12%] |
|  | Co-production [6.19%] | Barriers [0.50%] | - Lack of connection between PCNs and VCSE sector [0.30%] - Power imbalance between communities and NHS [0.20%] |
|  |  | Enablers [5.69%] | - Agreement that if this essentially voluntary approach to coordinating care can be achieved, then it will stick, and probably more firmly and effectively than if mandated by legislation [0.61%] - Communities are a central part of the public health system and community-centered ways of working should be integral to whole system action to improve population health [0.55%] - Recognizing the protective and risk factors at a community level that affect people’s health, and how these interact with wider determinants of health [0.49%] - Community-settings can influence individual and group experiences and offer potential for change and improving wellbeing and social inequalities [0.47%] - Community approaches demonstrate that cohesion is most sustainably built from the ground up [0.39%] - Shifting mindsets and redesigning the system, aligned to building healthy, resilient, active, and inclusive communities [0.39%] - Co-production of solutions with communities, based on conversations with people about health and place [0.34%] - Place-based narratives facilitate connections between people, objects, and events in urban landscape [0.33%] - Pooling resources and budgets across geographical boundaries or service types to meet shared aims [0.32%] - Arts-based approach helps to break down barriers, fosters appreciation, and increases engagement [0.32%] - Functional integration of resources needed, for example, sharing information and digital assets [0.31%] - Potential for developing meaningful, cross-sector strategies on issues such as workforce [0.29%] - Well understood processes for contacting different services and drawing upon their input [0.29%] - Undertaking insight work with communities especially those seldom heard [0.24%] - Local context is important as there is no one-size-fits-all [0.20%] - Communities differ significantly [0.15%] |
|  | Inter-professional relationships [1.87%] | Barriers [1.05%] | - Understanding of accountabilities and responsibilities between statutory partners needs to be improved [0.48%] - NHS architecture based on organizations working relatively autonomously rather than collaboratively [0.46%] - Differences in language [0.11%] |
|  |  | Enablers [0.82%] | - Right skills, capacities, and resources may need to be brought together from across systems [0.30%] - Improving communications in relation to program delivery and impacts [0.24%] - Ability and willingness to share information [0.15%] - Potential for learning between sectors [0.13%] |
| Costs  [9.52%] | Cost effectiveness [6.92%] | Barriers [4.69%] | - Direct non-medical costs less often included in cost analysis including costs associated with public health intervention such as developing media campaign, advertising, training, materials, and peer support events [1.04%] - Challenging financial position of some organizations made it harder for local systems to take collective responsibility for resources due to concerns about sharing financial risk with organizations in deficit [1.02%] - Organizations experienced difficulties in attracting funding or interest from social prescribing commissioners and needed to find alternative funding sources [0.73%] - Health and care providers face longstanding financial and operational challenges such as high level of staff vacancies in NHS and social care [0.47%] - Community focus on long-term impact, but short-term financial and political priorities drive system [0.46%] - Health Boards, Local Authorities and IJBs need joint understanding of respective financial positions [0.46] - Annual nature of funding creates uncertainty for projects [0.27%] - Differences in spending power and resources [0.20%] |
|  |  | Enablers [2.23%] | - Recognition of cost to partner organizations in terms of staff time to provide insight, take part in advisory groups and arrange for communities to participate in engagement activities [0.60%] - Payments to support participation from more diverse groups and help recognize value that organizations place on people contributing their experiences, knowledge, and skills [0.56%] - Cost analysis to consider costs incurred to develop and implement intervention including direct costs, indirect costs, and intangible costs [0.46%] - Good practice for organizations to have written policy giving details about reimbursing and paying people [0.35%] - Making best/sustainable use of resources/NHS resources is essential [0.26%] |
|  | Cost savings [2.60%] | Barriers [1.33%] | - Interventions may incur cost for participants, for example, outdoor clothing that could be provided for free [0.50%] - VCSEs in England report inadequate reimbursement and are forced to support costs through own budgets [0.47%] - Challenging nature of financial savings targets [0.22%] - Resources are a key challenge [0.14%] |
|  |  | Enablers [1.27%] | - Mapping out all funding streams across health and social care to ensure better alignment of funding and aid partners to take more strategic approach to deploying collective resources [0.60%] - Investing in community approaches generates greater impact for existing spend and saves money longer-term [0.35%] - Developing resources, such as training materials, that could be used on wider level post funding [0.32%] |
| Evidence and evaluation  [16.38%] | Evaluation methods [4.41%] | Barriers  [3.67%] | - Limitations and gaps in robust evaluation of community initiatives including how they were selected, what local need they met, how financially sustainable they were and what would have happened without them [1.04%] - Community approaches and initiatives required to demonstrate own worth according to measures not set up to recognize their value; value best captured qualitatively yet metrics are quantitative [1.02%] - Economic evaluations comparing costs and consequences of public health interventions with existing interventions usually require randomized controlled trial (RCT) approach [0.79%] - Researchers argue that social prescribing initiatives are not suitable for RCT approaches to cost effectiveness [0.50%] - Difficulties involved in undertaking research in the cultural sector [0.32%] |
|  |  | Enablers [0.74%] | - Qualitative evidence needs combination of semi-structured interviews or focus groups, participant observation and retrospective assessment [0.45%] - Monitoring and evaluation progress in achieving proposals [0.19%] - Evaluating impact and outcomes [0.10%] |
|  | Findings [4.82%] | Barriers [3.60%] | - Limited evidence that community infrastructure can reduce loneliness by being a point of contact, creating paid and volunteering opportunities, and encouraging social interaction [0.98%] - Limited evidence that social inclusion can be enhanced via green, blue, and public space interventions, community organizations and community-led social enterprises [0.98%] - Limited evidence as to how higher levels of social capital relate to improved lifestyle in terms of health [0.49%] - Limited evidence that social prescribers can remove barriers that stop patients from participating [0.46%] - Lack of evidence for long term effects of intervention; effects can dissipate relatively rapidly [0.45%] - Results remain inconclusive despite comparatively rich and deep data [0.32%] |
|  |  | Enablers [1.22%] | - Medium evidence that effective community infrastructure and social capital can lead to positive social outcomes including reduced loneliness, homelessness, offending rates and pressure on frontline services [0.70%] - Strong evidence that community infrastructure, strong social connectedness and social capital can have positive impact on community resilience and increased wellbeing [0.52%] |
|  | Focus of evidence [4.63%] | Barriers [3.10%] | - Existing metrics tend to have narrow focus on outcomes and expenditure that reflect silos of services rather than wider complex system and overarching outcomes for people as they move between those services [1.05%] - Whilst individual projects currently demonstrate benefits and impacts of their individual services, difficult to describe this at program or national level due to wide variation of projects funded [1.02%] - Many national datasets are inconsistent, published data on length of hospital stay of low quality, and no national data on NHS spending out of hospital [0.70%] - Community approaches are pluralistic, often small-scale and rooted in local context, but policymakers seek uniform and scalable approaches [0.34%] - Differences in metrics culture; NHS focus on backlog reduction [0.29%] |
|  |  | Enablers [1.53%] | - Economic analyses need to go beyond quality-adjusted life-years (QALY) to take account of all stakeholders and not just the NHS or health system [0.70%] - Evaluation needs to be built in from start, invest time in considering program aims to determine right evaluation methodology to make robust case to funding bodies [0.51%] - Data drawn from user experience needs to be placed on an equal footing with operational data at systems’ level [0.32%] |
|  | Future research [2.52%] | Barriers [1.41%] | - Formal structures and mechanisms put in place by legislation have yet to be fully tested [0.71%] - Significant gaps in data at interface between health and social care at national level [0.70%] |
|  |  | Enablers [1.11%] | - Consistent approach needed to collect range of data, including financial and economic costs, personal impacts, delivery models, demographics and outcomes relating to social determinants of health [0.70%] - More robust reporting arrangements with emphasis on demonstrating impact of projects on people [0.25%] - Important to assess and measure the right things [0.16%] |
| Integration of care  [20.53%] | Concept of integration [5.10%] | Barriers [3.34%] | - ICSs differ in terms of complexity and extent of joint working that existed prior to ICSs being established [0.50%] - PCNs developed at pace risk being implemented without understanding community health and care needs [0.47%] - Framework for community-based health and social care integrated services needs to be developed [0.44%] - No single ICS model, and each area is developing differently according to local circumstances [0.43%] - ICSs at early stage of development even in areas that were frontrunners [0.41%] - ICSs vary widely in size in terms of populations and number of organizations involved [0.40%] - IJBs should be empowered to use their resources to better meet local population needs [0.40%] - Need clear and shared understanding among local communities about what integrated care is [0.29%] |
|  |  | Enablers [1.76%] | - Creating opportunity to rebalance partnership between NHS and local authorities with both parties having equal influence on decision-making [0.46%] - Review of governance around ICF to ensure appropriate scrutiny arrangements in place for decisions made by RPBs [0.37%] - Local government and NHS broadly in agreement on overall aims of ICSs as set out by central government [0.34%] - NHS England consulted extensively in designing ICSs and widely supported by majority of stakeholders [0.33%] - Social value sector investment and delivery [0.15%] - Regionalisation and integration [0.11%] |
|  | Effectiveness of integrated care [8.09%] | Barriers [4.86%] | - Variation in ways that ICSs work include number of joint posts between organizations, shared commissioning practices, mutual scrutiny arrangements, local priorities and who leads on implementation [1.06%] - Regulators and national bodies slow to align how they work with ICSs particularly in way that regional teams of NHS England and NHS Improvement relate to NHS commissioners and providers [1.01%] - Some ICSs enjoy relative overall stability, but others experience financial and/or performance challenges [0.44%] - Challenges faced by NHS and care providers exacerbated by effects of pandemic [0.40%] - Improved strategic planning and commissioning arrangements needs to be put in place [0.39%] - More power and resources need to be given to local communities rather than held by central government or public services [0.36%] - Primacy of place but little focus on place in national policy [0.29%] - People at different levels of understanding about NHS structures [0.26%] - Further NHS support needed to help systems achieve [0.24%] - Governance of STPs remains in state of flux [0.21%] - ICSs may be too focused on health services [0.20%] |
|  |  | Enablers [3.23%] | - Trade-offs between treatment and preventative activity well established in health policymaking and optimism that ICSs will help to move balance between two toward more preventative activity [0.62%] - Access to social connection through local community groups or peer mentoring provide a more sustainable way of tackling issues that lead people to multiple visits to GPs and A&E [0.58%] - ICSs that are further ahead often have history of positive working relationships strengthened by regular face-to-face meetings and focused development [0.49%] - To support integration agenda, Welsh Government made education sector, local authority housing and housing associations statutory members of RPBs [0.48%] - Relationships between NHS and local authorities appears to be improving, with population health and its determinants featuring more strongly [0.46%] - ICSs report working more collaboratively to manage finances and performance across the system [0.31%] - Need clear and shared understanding among local communities about what integrated care is [0.29%] |
|  | Impact of integration [7.34%] | Barriers [4.06%] | - Growing issue about how load bearing ICSs should become and whether they should take on responsibility for quality and financial performance as opposed to planning and implementing transformation of care [1.09%] - Work cannot be seen as preserve of patient experience and public engagement teams alone and should be fundamental to all areas of work across ICSs, place-based partnerships, and organizations within them [0.94%] - In a rapidly changing environment with large-scale NHS structural reform within a pandemic-influenced world, risk that resultant structures will be out of touch with communities [0.82%] - Intense demands in managing recovery from Covid against backdrop of cost-of-living crisis, rising demand across services, profound workforce challenges and funding restraints [0.81%] - NHS focus on current performance rather than longer term population health management [0.40%] |
|  |  | Enablers [3.28%] | - Need to build on resources that exist locally including VCSE sector organizations, patient-participation groups and patient leaders already engaging with people and communities over wide range of issues. [0.70%] - Better integration of health and care services can help to remove barriers to collaboration, reduce duplication, create economies that increase cost-effectiveness, and improve patient experience [0.62%] - Access to social connection through local community groups or peer mentoring provide a more sustainable way of tackling issues that lead people to multiple visits to GPs and A&E [0.56%] - Creating opportunity to rebalance partnership between NHS and local authorities with both parties having equal influence on decision-making [0.46%] - Building people’s knowledge of how different parts of health and care system fit together and where their input will make a difference [0.40%] - Shifting mindsets and redesigning the system, aligned to building healthy, resilient, active, and inclusive communities [0.39%] - Social value sector investment and delivery [0.15] |
| Professional roles  [13.63%] | Community stakeholders [4.16%] | Barriers [0.85%] | - Lack of health professional understanding of reach of voluntary sector and its connectivity to communities [0.49] - More power and resources need to be given to local communities rather than held by central government or public services [0.36%] |
|  |  | Enablers [3.31%] | - Need to build on resources that exist locally including VCSE sector organizations, patient-participation groups and patient leaders already engaging with people and communities over wide range of issues [0.70%] - Recognition of people’s contributions beyond financial means including receiving acknowledgement in writing, support to develop skills and experience, and seeing improvements made because of their input [0.70%] - Whole systems approach responds to complexity through ongoing, dynamic and flexible way of working that enables local communities and other stakeholders to share an understanding of reality of challenge [0.70%] - Ongoing consultation with community stakeholders and relevant partners from health and third sectors is critical; organizations should invest time in developing these relationships [0.51%] - Local partners need to come together to develop co-ordinated services focused on needs of communities [0.33%] - Communities have wealth of knowledge and assets within themselves [0.22%] - Opportunities for social care commissioners and community providers [0.15%] |
|  | Employment and training [2.65%] | Barriers [1.78%] | - Staffing gaps that have developed and have become entrenched across health and care partly explain why recent staff absences due to COVID-19 have been so detrimental to NHS performance [0.85%] - Consistent approach to recruiting, training, and supporting range of people in different roles to help ensure knowledge, skills, and confidence to contribute effectively [0.56%] - No national NHS workforce strategy since 2003 [0.22%] - Staff recruitment and retention [0.15%] |
|  |  | Enablers [0.87%] | - Enthusiastic and committed staff who work well together, learn from each other, and form cohesive, team approach around service user [0.34%] - Share existing expertise through targeted training for other practitioners wanting to work in this space [0.34%] - Widespread agreement on skills needed to achieve progress [0.19%] |
|  | Leadership [3.53%] | Barriers [1.72%] | - Risk that if ICS leaders are not given sufficient time and space, they will not be able to deliver the radical changes to health and care services that the pandemic has demonstrated are needed [0.89%] - Leaders face competing demands on their time and priorities and sometimes struggle to devote time to ICS [0.43%] - Worries about the pipeline of future leaders of ICSs [0.25%] - Differences in leadership style [0.15%] |
|  |  | Enablers [1.81%] | - Collaborative leadership and building relationships with focus on shared and collaborative practice [0.33%] - Continuity of leadership and longstanding and respected leaders identified as key enablers of change [0.33%] - Leaders need to create the right culture in day-to-day meetings and communications with staff [0.32%] - Leaders in ICSs have adapted behaviors to lead using a facilitative and enabling approach [0.30%] - System leaders in social care sector are innovating to plug gaps in government support [0.29%] - Bold leadership to adapt radical approaches to reduce health inequalities [0.24%] |
|  | Link worker role [0.79%] | Barriers [0.43%] | - Loss of social support when an intervention comes to an end can be challenging for participants [0.43%] |
|  |  | Enablers [0.36%] | - Link workers need to identify and create links with individuals who do not traditionally access interventions [0.36] |
|  | Professional identity [2.50%] | Barriers [1.87%] | - Public health decision makers faced with limited resources must routinely make decisions about how to prioritize public health problems and choose among alternative interventions [0.83%] - Uncertainty about how experiences and insights of those leading primary care service at neighborhood level inform system-level planning and strategy [0.70%] - Variation in who the chairs and leads of STPs feel they are accountable to [0.34%] |
|  |  | Enablers [0.63%] | - Focus on senior-level buy-in to ensure project can develop with cross-sector support [0.28%] - Staff who are experts in their field, highly experienced and specialists, who can effectively support services users with complex needs [0.35%] |
| Service user factors  [21.18%] | Accessing care [9.05%] | Barriers [3.51%] | - Proximity to participants, public transport links, and public access all have bearing on whether participants continue post-intervention; participants need help to identify places they can access [0.91] - Promotion of inclusivity compromised by physical accessibility of properties, cost of transport or tickets, and lack of representation and training in workforce [0.74%] - Deprivation and poverty can prevent people taking part in activities unless financial support and resources in place that enable involvement [0.65%] - Labels and language particularly around mental health may have negative connotations or stigma attached [0.49%] - Barriers to cultural participation include cost, timings, location and lack of information [0.42%] - People need to be active participants in all efforts to improve their health and wellbeing [0.30%] - Social context can act as a barrier to participation [0.12%] |
|  |  | Enablers [5.54%] | - Being creative with referral pathways, individuals may find it easier to refer themselves or avoid dealing with professionals for various reasons including negative past experiences with health/social care services [0.70%] - Auditing and monitoring participation of certain groups to help identify gaps in engagement requiring attention and support staff to promote involvement of people more representative of population [0.64%] - Targeting resources and approaches at ethnic minority groups to provide additional support that some members will need because of the health disparities they experience [0.55%] - Using accessible venues, making reasonable adjustments or specific effort to ensure disabled people, autistic people and people with a learning disability can participate [0.55%] - Working with local community, third sector and voluntary organizations can be a route to engaging with people who might benefit from interventions [0.47%] - Health literacy levels and language should be relatable rather than using NHS terms and acronyms [0.45%] - Adopting single point of contact for a project so all partner organizations can refer to it in consistent manner [0.37%] - Signposting to local voluntary groups provides continuity for participants from activity and social perspective [0.37%] - Practical considerations to ensure different people can take part by making activities accessible [0.32%] - Asking people what form of recognition they would value and would support them to take part [0.30%] - Engaging with intended audience from outset shapes project to suit their needs appropriately [0.30%] - Flexible delivery allows different levels of engagement for different lengths of time [0.28%] - Social context can facilitate participation [0.12%] |
|  | Person-centered ethos [6.36%] | Barriers [1.64%] | - Public involvement left until last moment without enough time to carry out fair and proportionate exercise when public could and should have been involved earlier or to a greater extent [0.92%] - Traditional ways of working involve looking at ”what is the matter” with an individual, not “what matters” to them; need to move to a more holistic approach [0.72%] |
|  |  | Enablers [4.72%] | - Need to ask the right questions; rather than asking about people’s experiences of individual services, ask questions focused on partnership working and coordination of services, and people’s experience of these [0.99] - Individuals that do not traditionally access intervention may be part of other community groups or networks [0.50%] - Ensuring that those with greatest needs and poorest health outcomes are engaged with and listened to, not just those who speak the loudest [0.45%] - Creative and flexible approaches, that could be piloted and refined to meet needs of individual service users [0.36%] - NHS organizations need to involve public alongside duties to act effectively, efficiently, and economically [0.36%] - Participatory methods navigate complex socio-economic challenges and strengthen legitimacy of decision-making [0.36%] - Adequate resources to provide intensive and tailored support over a long period of time to individuals [0.34%] - Staff having the time and resources to support individuals in a tailored way [0.34%] - Voice and experience of people and communities needs to be at heart of health or care organization [0.32%] - Opportunity to develop genuinely joined-up, personalized care [0.20%] - Co-creation of interventions and programs with end users [0.19%] - Focus on both direct and indirect support for carers [0.18%] - Informal and safe environment [0.13%] |
|  | Service user outcomes [5.77%] | Barriers [2.57%] | - Responsibility to keep people involved safe; being asked repeatedly to go back over bad experiences so professionals can learn how to improve services causes distress and increases lack of trust [0.90%] - People from socio-economically disadvantaged groups and Black, Asian, and minority ethnic communities tend to be under-represented in cultural activities [0.71%] - Carers and representatives of service users need better support to enable full involvement in integration [0.49%] - Effective working relationships with carers, service users and local communities need to be improved [0.47%] |
|  |  | Enablers [3.20%] | - Harnessing power of communities and recognizing and building on existing community assets would bring greater connection and trust and help identify and deliver local solutions to tackling health inequalities [0.70%] - Skills acquired should have some universality in daily life (for example meditation, mindfulness, journaling, team building, leadership skills) to be applied to everyday stressors, situations and relationships [0.70%] - Involving people in decision making, alongside supporting them with resources and wider social infrastructure, can enable community action to improve wellbeing and resilience locally [0.58%] - Community power can enhance the engagement of those communities least likely to access mainstream health services and boost trust and confidence [0.45%] - Successful intervention supports lasting change or enduring benefit for participants [0.28%] - Building relationships based on trust, particularly with marginalized groups [0.25%] - Effective implementation of cohesive pathways of care for service users [0.24%] |
